# Cortical organoid-derived models of the melanoma brain metastatic niche enable prioritization of cancer-targeting drugs

**DOI:** 10.1016/j.crmeth.2025.101236

**Published:** 2025-11-14

**Authors:** Kim Krieg, Silvia Materna-Reichelt, Tobias Naber, Fatima-Zahra Rachad, Pia Kauven, Arjen Weller, Undine Haferkamp, Annika Wittich, Andrea Zaliani, Marcel S. Woo, Mark Walkenhorst, Malte Siegmund, Jann Harberts, Robert Zierold, Robert Blick, Christian Conze, Patricia Muschong, Dominik Miltner, Manuel A. Friese, Mario Mezler, Heiko Siegmund, Katja Evert, Susanne Krasemann, Nataša Stojanović Gužvić, Christoph A. Klein, Melanie Werner-Klein, Joachim Wegener, Ole Pless

**Affiliations:** 1 Fraunhofer Institute for Translational Medicine and Pharmacology ITMP, Discovery Research ScreeningPort, 22525 Hamburg, Germany; 2 Fraunhofer Institute for Toxicology and Experimental Medicine ITEM-R, Division of Personalized Tumor Therapy, 93053 Regensburg, Germany; 3 Fraunhofer Institute for Electronic Microsystems and Solid-State Technologies EMFT, Cell-based Sensor Technology, 93053 Regensburg, Germany; 4 Institute for Analytical Chemistry, Chemo- & Biosensors, University of Regensburg, 93053 Regensburg, Germany; 5 Institute of Neuroimmunology and Multiple Sclerosis, University Medical Center Hamburg-Eppendorf, 20251 Hamburg, Germany; 6 Center for Hybrid Nanostructures, University of Hamburg, 22761 Hamburg, Germany; 7 Technology Platform Microscopy and Image Analysis (TP MIA), Leibniz Institute of Virology, 20251 Hamburg, Germany; 8 AbbVie Deutschland GmbH & Co. KG, 67061 Ludwigshafen, Germany; 9 Institute for Pathology, University of Regensburg, 93053 Regensburg, Germany; 10 Institute for Neuropathology, University Medical Center Hamburg-Eppendorf, 20246 Hamburg, Germany; 11 Experimental Pathology Core Facility, University Medical Center Hamburg-Eppendorf, 20246 Hamburg, Germany; 12 Experimental Medicine and Therapy Research, University of Regensburg, 93053 Regensburg, Germany

**Keywords:** cortical organoids, melanoma brain metastases, disseminated cancer cells, preclinical cancer model, drug discovery, assay development, high-throughput screening, personalized medicine, XPO1, selinexor

## Abstract

Effective systemic therapies against brain metastases are severely limited. To understand and target vulnerabilities of human metastases in a brain niche context, we developed reproducible melanoma brain metastasis (MBM) models for metastasis-integrating drug screening. We co-cultured A375 melanoma cells or tumor regional lymph node-derived disseminated cancer cells (DCCs) in close proximity with human induced pluripotent stem cell-derived cortical organoids (hCOs). In these, RNA sequencing revealed an upregulation of metastasis-associated features. First, A375 cells and DCCs were screened against an anti-cancer library containing 315 compounds. Hits were ranked by neurotoxicity, central nervous system permeation, and anti-DCC efficacy. Only a minority of hits effectively targeted A375-MBMs, with the first-in-class XPO1 inhibitor selinexor emerging as top hit. Selinexor also demonstrated efficacy in DCC-MBM models and low toxicity on hCOs, suggesting a promising therapeutic window in clinically applied doses. Collectively, the MBM model provides a tool for identifying candidate therapies counteracting metastatic progression.

## Highlights

• We develop melanoma brain metastasis (MBM) models using human cortical organoids
• MBM models can be applied to identify therapies targeting metastatic progression
• Compound screening with MBM models nominates selinexor as a therapeutic candidate

## Motivation

There is a current lack of human models to identify effective therapies targeting brain metastases. In this work, we therefore sought to develop a robust method to study and target vulnerabilities of human metastases in the context of brain niche tissue. We aimed to integrate the high-throughput screening-compatible model into workflows for the identification of candidate therapeutics that counteract metastatic progression.

## Graphical abstract

**Figure:**
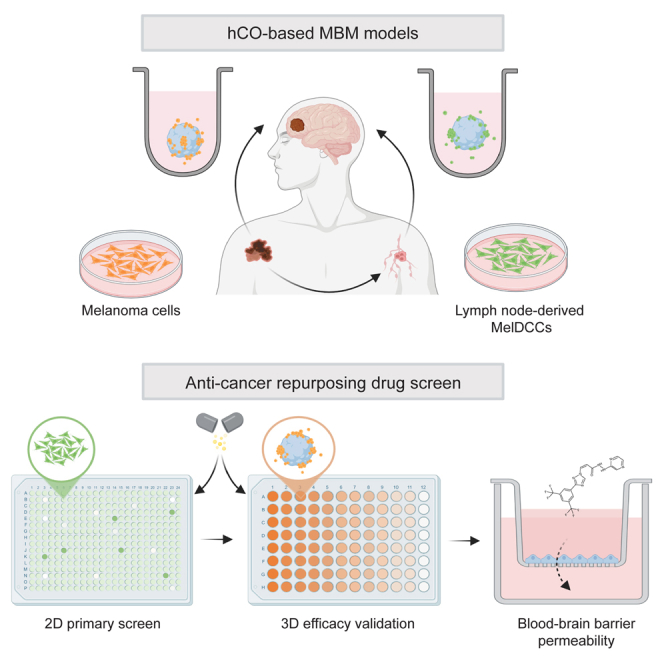

## Teaser

Krieg et al. develop melanoma brain metastasis models based on cortical brain organoids. They demonstrate the potential for therapeutic applications, screening a library of anti-cancer compounds and identifying the XPO1 inhibitor selinexor as a promising candidate that demonstrates effectiveness against cancer cells while maintaining low toxicity.

## Introduction

Brain metastasis (BrM), the dissemination and growth of cancer cells to the central nervous system (CNS), occurs in at least 20% of cancer patients and imposes a significant clinical challenge.^1^ Currently available treatment options include surgery, radiation therapy, and systemic therapies, primarily immune checkpoint inhibitors and targeted therapy, providing benefits to subgroups of patients. However, systemic therapies are frequently associated with limited efficacy and fail to reliably prevent relapse and long-lasting disease progression.^2^^,^^3^^,^^4^^,^^5^ Consequently, the use of predictive preclinical BrM models to target therapeutic vulnerabilities with anti-cancer agents could pave the way for prospective clinical trials that improve understanding of efficacy and CNS safety. Thus, several studies have developed *ex vivo* drug screening platforms utilizing mouse organotypic cultures, patient-derived and xenograft models.^6^^,^^7^^,^^8^ In melanoma patients, the lifetime risk of developing BrM stays notably high up to approximately 40%.^9^^,^^10^^,^^11^ During the early stages of metastasis, melanoma cells undergo phenotypic and molecular alteration and spread primarily to regional lymph nodes, which act as key exit routes for systemic dissemination via the bloodstream enabling melanoma cells to extravasate into the brain parenchyma or other distant organs.^12^^,^^13^^,^^14^^,^^15^^,^^16^ Importantly, quantitative assessments of disseminated cancer cells (DCCs) in sentinel lymph node biopsies of melanoma patients allow for prediction of disease progression.^17^^,^^18^ Thus, tumor regional lymph nodes represent reservoirs of the earliest metastatic seeds, providing an accessible and clinically relevant source of cells for preclinical drug testing (S.M.-R., M.W.-K., C.A.K., K. Weidele, C. Werno, and S. Treitschke, unpublished data). Metastatic organ colonization, the progression from solitary cancer cells to micrometastases, is considered the limiting step in the metastatic cascade.^19^^,^^20^ During this critical phase, DCCs utilize phenotypic plasticity to adapt to and thrive within the foreign tumor microenvironment (TME) of the metastatic sites, making early colonization a pivotal therapeutic window. Bidirectional communication between invading DCCs and native cellular and non-cellular components has critical impact on metastatic growth, treatment response, and resistance.^21^^,^^22^^,^^23^^,^^24^^,^^25^ This highlights the CNS microenvironment as an important component to consider in the development of novel therapeutic strategies targeting metastatic cancer cells in the brain. Human cortical organoids (hCOs) are self-organizing CNS-like structures derived from human induced pluripotent stem cells (hiPSCs). They contain neurons and astrocytes, which support neural function and can modulate inflammatory responses.^26^^,^^27^ Such hCOs have been utilized as scaffolding host tissues for several primary tumor cells, such as small-cell lung cancer and breast cancer cells, to mimic BrM.^28^^,^^29^^,^^30^ In this study, we implemented and validated a scalable platform of melanoma brain metastasis (MBM) models by using hCOs as a niche tissue for the colonization of melanoma cells and patient-derived DCCs. Based on this model, an anti-cancer drug repurposing library was evaluated with respect to metastasis regression *in situ*, enabling the identification and validation of biologically relevant drug candidates in a human brain niche-specific context.

## Results

### Establishment of a scalable platform for cortical organoid and BrM model generation

**Figure 1:**
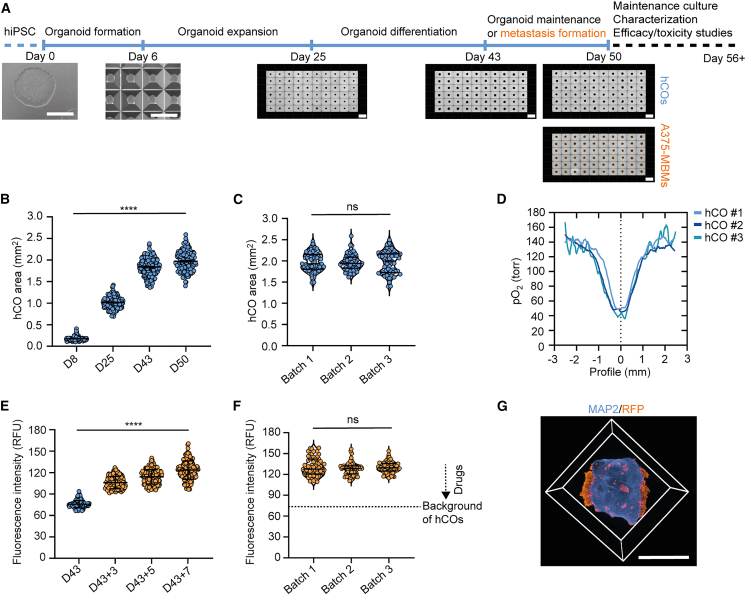
Robust generation of human cortical organoids and melanoma brain metastases in a screening-compatible format (A) Schematic overview and microscopic images illustrating stages of hCO differentiation and metastasis formation using fluorescent labeled A375 melanoma cells (A375-MBMs) along the experimental timeline. Scale bars in both left images represent 1 mm; in 96-well plate overview the scale bars represent 2 mm. (B) Increase of hCO area during differentiation (*n* ≥ 175 hCOs from 3 independent batches per time point). Data are presented as mean ± SEM. Statistical analysis was performed using one-way ANOVA, followed by Tukey’s multiple comparisons test. ∗∗∗∗*p* < 0.001. (C) Consistent mean hCO area at day 50 (*n* ≥ 58 hCOs per batch). Data are presented as median and lower/upper quartile. Statistical analysis was performed using one-way ANOVA, followed by Tukey’s multiple comparisons test. ns, not significant. (D) Reproducible oxygen profiles through hCOs after 6 h of attachment to the sensor foil (*n* = 3 hCOs from independent batches). The dashed vertical line marks the center of hCOs. (E) Time course analysis of A375-MBMs fluorescence intensity during 7 days of colonization (*n* ≥ 176 hCOs from 3 independent batches). Data are presented as mean ± SEM. Statistical analysis was performed using one-way ANOVA, followed by Tukey’s multiple comparisons test. ∗∗∗∗*p* < 0.001. (F) Consistent mean fluorescence intensity of A375-MBMs after 7 days (*n* ≥ 58 per batch), offering a stable assay window to test drugs reducing viability of colonized A375 cells. Data are presented as median and lower/upper quartile. Statistical analysis was performed using one-way ANOVA, followed by Tukey’s multiple comparisons test. ns, not significant. (G) Three-dimensional rendering of A375-MBMs visualized by whole-mount staining and light-sheet microscopy for A375 cells (RFP) and neuronal tissue (MAP2). Scale bar represents 1 mm. See also Figure S1.

To generate reproducible hCOs suitable for screening applications, we adapted a guided differentiation protocol^31^ and established a standardized culture and compound treatment procedure (Figure 1A). hCOs generated from wild-type hiPSCs (WISCi004-B) were uniformly aggregated in microwells until day 6, after which they were individually transferred to 96-well round-bottom ultra-low attachment plates for further differentiation (Figures 1A; S1A). Throughout the culture period in 96-well plates, we monitored the organoid morphology of representative batch plates through bright-field imaging and spheroid detection analysis (Figure S1B). hCOs derived from hiPSC line WISCi004-B (Figure 1B), but also ZIPi013-B and HHUUKDi009-A (Figure S1C), exhibited progressive growth, reaching a similar area of approximately 2 mm^2^ (1.970 ± 0.002 mm^2^), with WISCi004-B demonstrating the lowest degree of intra-batch variability. Notably, these 50-day-old hCOs can be generated with consistent organoid areas and roundness both within individual batches and across batches, with a mean coefficient of variation of 11.56% ± 1.13% (area, Figure 1C) and 10.45% ± 0.41% (roundness, Figure S1D). Additionally, measurements of the partial oxygen pressure in randomly selected hCOs from different batches revealed similar profiles and adequate mean oxygenation above 45 torr, even within the central tissue core (Figures 1D and S1E).^32^^,^^33^^,^^34^^,^^35^ These findings demonstrate the simultaneous production of reproducible and physiologically active batches of hundreds of hCOs. To initiate and visualize MBMs, we used the human melanoma cell line A375 stably transduced with a red fluorescent protein (RFP) tdTomato reporter (Figure S1F). A375 cells were added as a single-cell suspension to each 43-day-old hCO and co-cultured for 7 days (day 43 + 7, referred to as A375-MBMs), followed by characterization or compound treatment (Figure 1A). During this co-culture period, we selectively monitored the fluorescence intensity of cancer cells within the hCO area, representing colonized cancer cells (Figure S1G). The fluorescence intensity increased progressively over time, indicating significant adherence and proliferation of A375 cells in the hCO tissue (Figure 1E). Importantly, a comparative analysis between batches showed minimal intra- and inter-batch variability, with a mean coefficient of variation of 7.92% ± 1.95% (Figure 1F). These data indicate a robust assay window, in which potent drugs may reduce the fluorescence intensity as an indicator of A375-MBM viability to baseline. Light-sheet microscopy of a cleared A375-MBM sample, followed by three-dimensional rendering, revealed multiple RFP+ A375 colonies of various sizes and shapes distributed across the hCO, co-stained for the neuronal marker MAP2 (Figure 1G). These data suggest that the melanoma cells exhibit pronounced adhesion, infiltration into, and colonization of the neural parenchyma.

### Characterization of brain parenchyma and melanoma BrM-like signatures

**Figure 2:**
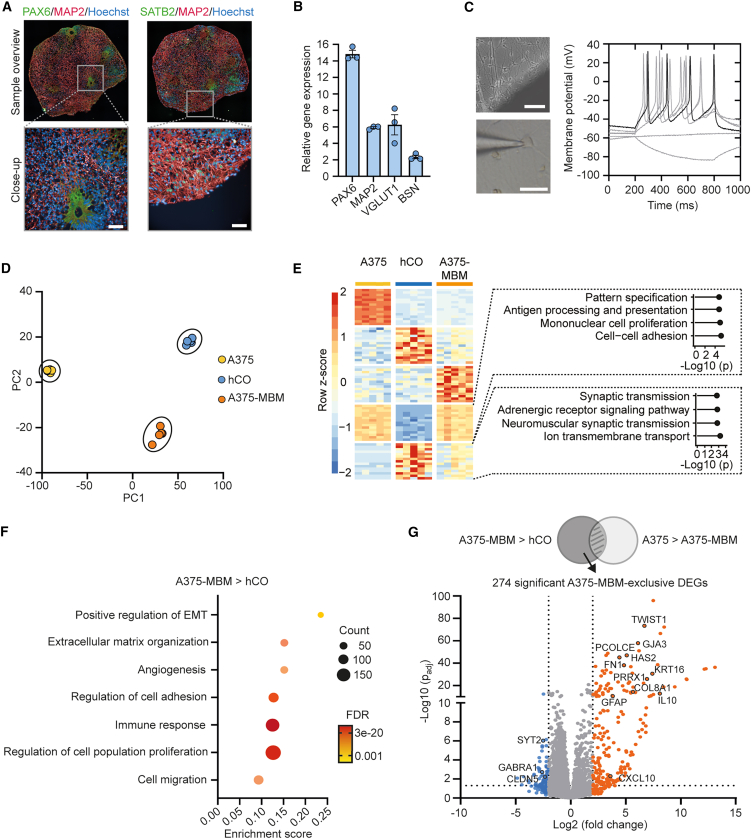
Characterization of cortical organoids and their co-culture with A375 cells (A) Immunofluorescent confocal images of different hCO cell types stained for PAX6, MAP2, and SATB2. Images present sample overview or labeled sections with higher magnification. Scale bars represent 50 μm. (B) hCO gene expression profile of *PAX6*, *MAP2*, *VGLUT1*, and *BSN* (*n* = 3 independent batches). Data are presented as mean ± SEM. (C) Representative action potentials of outgrowing neurons from plated 50-day-old hCOs recorded during electrode polarization in current clamp mode. Black trace corresponds to rheobase value. Scale bar (upper) represents 100 μm, scale bar (lower) represents 20 μm. (D) Principal-component analysis plot of mRNA-sequencing data of A375 cells, hCOs, and A375-MBMs (*n* = 5 samples per condition). (E) Top 10 differentially expressed genes identified by mRNA sequencing in the following modules: only upregulated in A375, only upregulated in hCO, only upregulated in A375-MBM, upregulated in A375 and A375-MBM, and upregulated in hCO and A375-MBM. The heatmap shows row *Z* scores. Gene ontology (GO) term of the differentially expressed genes in A375 and A375-MBM and in hCO and A375-MBM is additionally shown. (F) GO term analysis of differentially upregulated genes in A375-MBMs in comparison to hCOs. FDR, false discovery rate. (G) Volcano plot representation of significantly up- and downregulated differentially expressed genes in A375-MBM in comparison to hCO, excluding genes that were differentially upregulated in A375 in comparison to A375-MBM, thereby predominantly expressed in A375 cells before hCO colonization. See also Figure S2.

To investigate the hCO cytoarchitecture, samples from different batches were sectioned for histochemical analysis. By day 50, the majority of cells throughout the parenchyma had differentiated into neuronal cells and strongly expressed the marker MAP2 (Figure 2A). hCOs did not fully reach postmitotic stages, since we identified neural rosettes with a population of neural progenitor cells (NPCs) expressing PAX6 (Figure 2A). Following directed neural development, hCOs developed cortical-like structures with both ventricular zones (Figure S2A) and cortical plate, including superficial cortical neurons expressing SATB2 (Figure 2A). Additionally, we performed RT-qPCR analysis and observed that the transcriptional identity of neural progenitors (*PAX6*) and neurons (*MAP2*), including glutamatergic neurons (*VGLUT1*) and synapses (*BSN*), was reproducible across differentiation batches (Figure 2B). Within the neural tissue (Figure S2B), we identified synapse-like structures (Figure S2C), and, for functional analysis, outgrowing neurons from post-seeded differentiated hCOs were accessible for patch-clamp techniques. Neurons exhibited passive membrane properties similar to published data of diverse hiPSC-based neuronal *in vitro* cultures (Figures S2D and S2E).^36^^,^^37^ Functional neurons were found across different batches as demonstrated by their AP trains with distinct in- and outflow currents and defined AP shapes (Figures 2C and S2F**–**S2I), i.e., type 5 neurons.^38^ We investigated the molecular changes associated with A375 colonization and performed bulk mRNA sequencing of five samples each of A375 cells prior to colonization, hCOs alone, and A375-MBMs. Principal-component analysis (PCA) revealed a strong clustering of the samples within the different groups (Figure 2D). In accordance, we detected strong neuronal signatures in hCOs and A375-MBMs as well as transcriptional signatures related to proliferation and adhesion in A375 cells and A375-MBMs that were absent in hCOs (Figure 2E). Next, we aimed to identify genes that were differentially expressed by co-culturing the A375 cells and hCOs. Therefore, we focused on differentially expressed genes (DEGs) in A375-MBMs compared to hCOs. Gene ontology (GO) term analysis confirmed an enrichment of genes involved in metastasis-associated processes, including “immune response” (GO:0006955), “extracellular matrix organization” (GO:0030198), and “positive regulation of epithelial-to-mesenchymal transition (EMT)” (GO:0010718) (Figure 2F). To identify genes that were exclusively differentially expressed in A375-MBMs, we omitted candidates that were predominantly expressed in A375 cells prior to brain organoid colonization. This analysis identified a total number of 274 DEGs, including 199 upregulated and 75 downregulated genes (Figure 2G). Among the most significant DEGs, we identified *TWIST1*, a key transcription factor inducing EMT (−Log10 (padj) = 73.45). In summary, we identified significant transcriptional changes associated with EMT (*TWIST1* and *PRRX1*), extracellular matrix (ECM) (*PCOLCE*, *HAS2*, and *FN1*), and immune response (*GFAP*, *IL10*, and *CXCL10*) as a result of brain organoid colonization.

**Figure 3:**
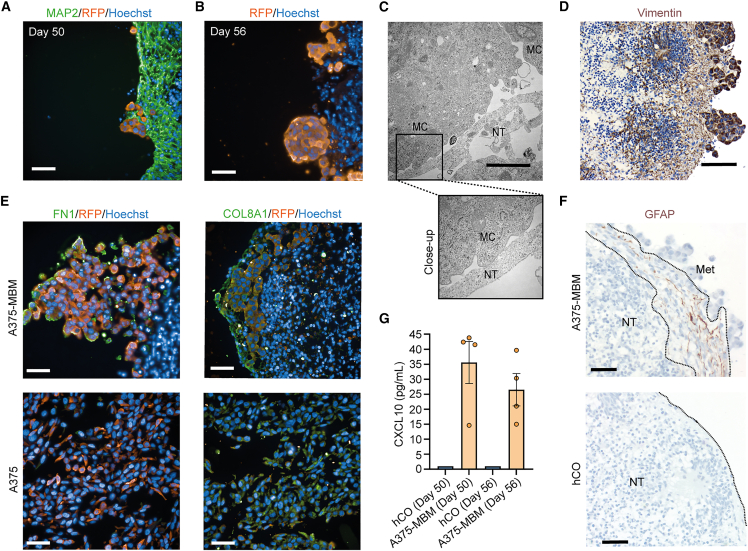
Validation of metastasis-like signatures in A375-MBMs (A) Immunofluorescent confocal image of A375-MBMs on day 43 + 7 (day 50) stained for MAP2 and RFP indicates tumor colony formation. Scale bar represents 50 μm. (B) Immunofluorescent confocal image of nodular metastasis outgrowth stained for RFP in prolonged A375-MBM cultures on day 43 + 13 (day 56). Scale bar represents 50 μm. (C) Ultrastructural analysis of metastasis-like structures shows close proximity between melanoma cells (MCs) and neural tissue (NT) at day 43 + 7 (day 50). Scale bar represents 2 μm. (D) Immunohistochemistry of A375-MBMs shows a strong Vimentin staining in the colonized melanoma cells at day 43 + 13 (day 56). Scale bar represents 100 μm. (E) Immunofluorescent confocal images of A375 before and after colonization (A375-MBM) at day 43 + 13 (day 56) demonstrate staining of extracellular matrix components FN1 and COL8A1 in metastases. Scale bar represents 50 μm. (F) GFAP-expressing cells in the interface of neural tissue (NT) and metastatic-like outgrowth (Met) in A375-MBMs and hCOs at day 43 + 13 (day 56) visualized by immunohistochemistry staining. Scale bar represents 50 μm. (G) Elevated CXCL10 concentration in the supernatant of A375-MBM at day 43 + 7 (day 50) or day 43 + 13 (day 56) compared to hCOs (*n* = 2 samples of each, two independent differentiations). Data are presented as mean ± SEM. Values for hCOs are under the lower limit of detection and were set to 1.

To assess the spatial relationship and distribution of metastatic A375 cells within the MBMs, we performed histological staining on paraffin-embedded samples. We observed a nested growth pattern, with RFP-expressing cancer cells forming nodule-like structures at the organoid surface, with more and bigger structures in prolonged A375-MBM cultures (day 43 + 13/56) (Figures 3A and 3B). A subpopulation of mostly single A375 cells penetrated the superficial organoid layers. Consistent with these data, ultrastructural analysis using transmission electron microscopy demonstrated direct contact sites between A375 and neuronal cells (Figure 3C). Notably, we detected besides the presence of Vimentin (Figure 3D) also a strong transcriptional and protein induction of the ECM component Fibronectin 1 (*FN1*; −Log10 (padj) = 37.97) and Collagen type VIII (COL8A1; −Log10 (padj) = 14.43) (Figures 2G and 3E), indicative of a mesenchymal phenotype of colonizing A375 cells. Furthermore, upregulation of GFAP (−Log10 (padj) = 10.66) suggests the presence of astrocyte-like cells (Figure 2F). In prolonged A375-MBM cultures (day 43 + 13/56), a population of astrocyte-like cells was observed in superficial layers, interspersed between melanoma cells and neurons, which were absent in the corresponding hCOs (Figure 3G). In addition, the expression of CXCL10, a chemokine associated with melanoma brain tropism,^39^ was increased in supernatant of A375-MBM models in comparison to the corresponding hCOs (Figure 3G).

### Drug repurposing strategy for MBMs

**Figure 4:**
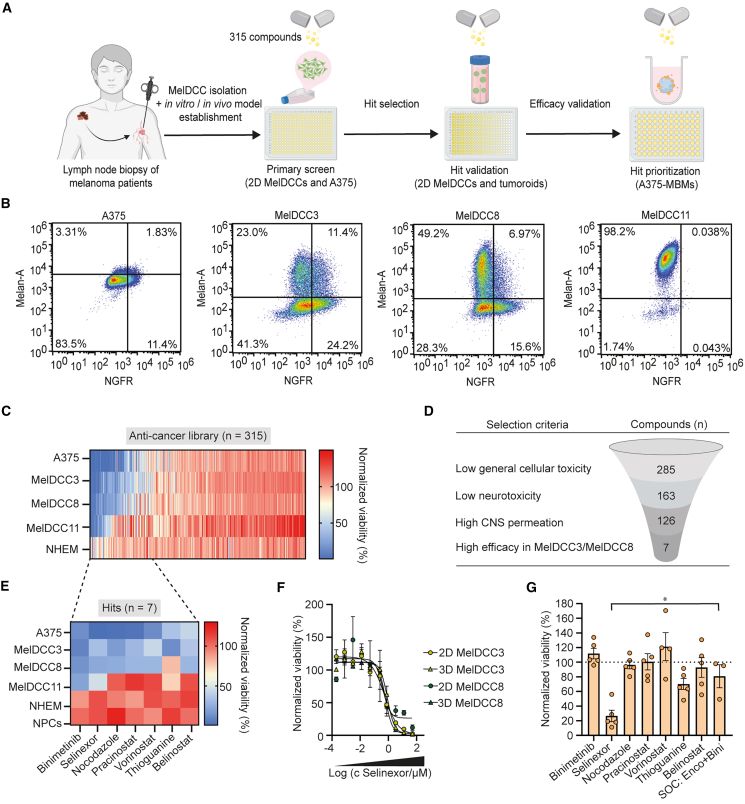
Anti-cancer drug screen and MBM validation strategy (A) Schematic overview of the drug repurposing screening strategy starting with the primary screen using 2D A375 cells and MelDCCs from patient’s lymph node specimen. (B) Flow cytometry analysis of MelDCC and A375 cells stained for Melan-A and NGFR receptor. (C) Heatmap of ATP-based cellular viability of A375, MelDCC3, MelDCC8, and MelDCC11 cells and NHEM control cell line after 5-day exposure to 315 anti-cancer drugs screened at 1 μM. Viability values are ranked from left to right based on their impact on the mean viability of all cell lines. Each data point is represented by one individual colored line. (D) Workflow outlining the criteria and steps for rational hit selection starting from 2D screening data. (E) Close-up of heatmap showing the sensitivity of the cell panel including neural progenitor cells (NPCs) toward selected compound hits. (F) Concentration-response titration of selinexor on ATP-based cellular viability of 2D and tumoroid (3D) cell cultures (*n* = 2–3 experiments per condition). Data are presented as mean ± SEM. IC_50_(2D MelDCC3): 0.83 μM; IC_50_(3D MelDCC3): 0.54 μM; IC_50_(2D MelDCC8): 0.6 μM; IC_50_(3D MelDCC8): 0.4 μM. (G) Fluorescence-based cellular viability of A375-MBMs treated with hit compounds at 10 μM for 72 h (*n* = 3–5 experiments). Data are presented as mean ± SEM. Statistical analysis was performed using one-way ANOVA, followed by Tukey’s multiple comparisons test. ∗*p* = 0.0179. See also Figure S3.

To identify clinically promising compounds targeting metastases with robust efficacy and established safety profiles, we conducted a drug repurposing screen using a focused library of 315 cancer-targeting compounds (all Food and Drug Administration approved or in clinical development; Figure 4A). We used GFP-labeled DCC lines derived from tumor regional lymph nodes of melanoma patients (MelDCC)^13^ or primary tumor (A375), representing the diverse, previously described differentiation phenotypes^40^ (Figures 4A and S3A). Normal human epidermal melanocytes (NHEMs) served as non-cancer controls. Given the importance of *BRAF* and *NRAS* driver mutations, we included BRAF-mutant (V600E: MelDCC3, MelDCC11, and A375) and NRAS-mutant (G12C: MelDCC8) lines. Differentiation phenotypes were confirmed by fluorescence-activated cell sorting analysis of Melan-A (melanocytic) and NGFR (neural-crest) expression (Figures 4B and S3B). While A375 and MelDCC11 represent undifferentiated and melanocytic phenotypes, respectively, MelDCC3 and MelDCC8 contain mixed phenotypes (melanocytic, neural crest-like, and undifferentiated). With this diverse melanoma panel at hand, the 315 compound anti-cancer library was screened at a concentration of 1 μM in 2D MelDCCs and A375 cells, recording viability values after 5 days of treatment (Figure 4C). Screening data of MelDCC11 and NHEMs were sourced from S.M.-R., M.W.-K., C.A.K., K. Weidele, C. Werno, and S. Treitschke (unpublished data) and re-analyzed. Using 10 μM bortezomib, a potent pan-proteasome inhibitor with high anti-cancer activity,^41^ as a positive control and 0.1% DMSO as vehicle, we reached *Z*′ scores ≥0.67 for each screening plate (Figure S3C), indicative of a high-quality screen,^42^ with high correlation between replicates (Figure S3D). Compounds were ranked for their strongest impact on mean viability on all cell lines tested (Figure 4C). We observed varying sensitivities to compounds between cell lines. MelDCC3, MelDCC8, and A375, which exhibit a more dedifferentiated phenotype based on their Melan-A and NGFR expression, were generally more responsive than the differentiated, melanocytic MelDCC11 line or NHEM cells. For rational hit selection, we first eliminated candidates exhibiting general cellular toxicity, characterized by <80% viability in control NHEM cells (Figure 4D). Next, we excluded compounds with neurotoxicity (<80% viability) based on previously published screening data and a high-quality drug repurposing library^43^ using hiPSC-derived NPCs.^44^^,^^45^ Considering the significant dependency of drug efficacy on blood-brain barrier (BBB) permeation, we further included physico-chemical properties into the selection process. Selected compounds presented a topological polar surface area value below 118, assuming that the charged atomic space primarily influences the permeation through the BBB.^46^ Finally, hits were characterized based on high efficacy (mean E_max_ ≥ 60%) to target the two most responsive MelDCC lines, MelDCC3 and MelDCC8. This process ultimately led to the nomination of seven compounds (Figure 4E): A MEK1/2 inhibitor (binimetinib), a XPO1 inhibitor (selinexor), a microtubule polymerization inhibitor (nocodazole), three HDAC inhibitors (pracinostat, vorinostat, and belinostat), and an antimetabolite (thioguanine). All candidates were confirmed in independent concentration-response titrations on MelDCC lines in 2D, but also 3D tumoroid culture, thereby increasing cancer tissue complexity (Figures 4F; S3E**–**S3J). To prioritize these validated hit compounds for their efficacy on BrM, we measured their cytotoxic effect in A375-MBM at a concentration of 10 μM for 72 h. We compared these results to a combination of BRAF and MEK1/2 inhibitors (encorafenib and binimetinib), one of the current standards of care in advanced melanoma^47^ (Figure 4G). Notably, we identified selinexor as inducing the most significant decrease in fluorescence-based A375-MBM viability (26.6% ± 7.9% remaining viability). Consequently, selinexor was selected as the top candidate targeting BrM for further investigations.

### The MBM platform confirms selinexor’s selective effect on metastasis regression through inhibition of nuclear export

**Figure 5:**
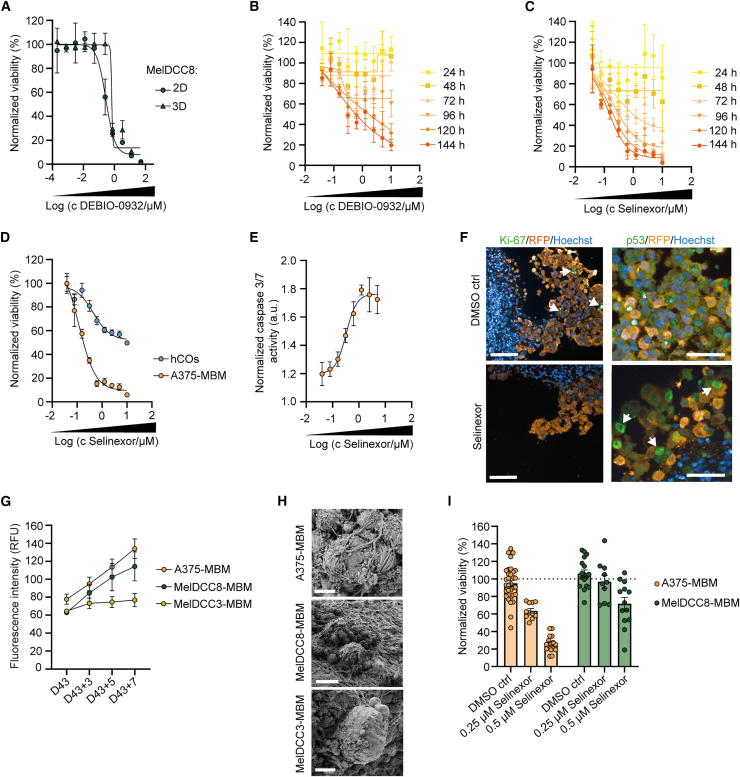
Selinexor selectively causes metastasis regression in A375- and MelDCC8-MBMs (A) Dose-dependent effect of DEBIO-0932 on ATP-based cellular viability of 2D and tumoroid (3D) MelDCC8 cell cultures after 5 days (*n* = 2 experiments per condition). Data are presented as mean ± SEM. IC_50_(2D): 0.65 μM; IC_50_(3D): 0.23 μM. (B) Dose- and time-dependent effect of DEBIO-0932 on fluorescence-based cellular viability of A375-MBMs (*n* = 3 technical replicates per condition). Data are presented as mean ± SD. IC_50_: 0.25 μM. (C) Dose- and time-dependent effect of selinexor on fluorescence-based cellular viability of A375-MBMs (*n* = 3 technical replicates per condition). Data are presented as mean ± SD. IC_50_: 0.13 μM. (D) Concentration response of selinexor in A375-MBMs and hCOs. Viability is assessed based on the fluorescence intensity or ATP-based 3D CellTiter-Glo assay after 6 days of treatment (*n* = 2 experiments per condition). Data are presented as mean ± SEM. (E) Concentration-dependent caspase-3/7 activity in A375-MBM after 6 days of selinexor exposure (*n* = 2 experiments). Data are presented as mean ± SEM. (F) Immunohistochemical confocal images of A375-MBMs treated with 0.25 μM for 72 h of cancer cells (RFP), proliferation marker Ki-67, and XPO1 target TP53. Scale bars represent 50 μm. (G) Time-course analysis of MelDCC3- and MelDCC8-MBMs compared to A375-MBMs during 7 days of colonization (*n* = 6 independent batches each). Data are presented as mean ± SEM. (H) Ultrastructural analysis of A375-, MelDCC8-, MelDCC3-MBMs demonstrates close proximity of colonized cancer and neuronal cells. Scale bars represent 10 μm. (I) Fluorescence-based cellular viability readout of MBMs treated with 0.25 and 0.5 μM selinexor for 144 h (*n* = 10–33 from at least 2 independent experiments). Data are presented as mean ± SEM. See also Figure S4.

Due to recent efforts in preclinical and clinical trials to repurpose selinexor for glioblastoma patients,^48^^,^^49^ we investigated in depth its efficacy and toxicity aspects with the MBM platform. As a reference compound, we selected the HSP90 inhibitor DEBIO-0932, due to its previously established efficacy against BrMs and good BBB permeability.^8^^,^^50^ In our experiments, DEBIO-0932 exhibited a defined concentration-dependent toxicity in 2D and 3D tumoroid MelDCC8 cultures (Figure 5A). In addition, fluorescence-based monitoring of A375-MBMs demonstrated DEBIO-0932’s potent anti-metastatic activity, with viability decreasing significantly to 19.7 ± 5.5% at 10 μM (IC_50_ of 0.25 μM) in a concentration-dependent manner over 6 days with daily compound replenishment (Figure 5B). Next, we investigated the selinexor response of A375-MBMs across a concentration range of 0.04–10 μM, spanning the maximal plasma concentrations of 0.65 μM detected in patients after oral administration.^48^^,^^51^^,^^52^^,^^53^ In comparison to DEBIO-0932, selinexor exhibited a faster onset and more potent reduction of A375-MBM (3.7% ± 1.6% at 10 μM; IC_50_ of 0.13 μM) (Figure 5C). Exposure of hCOs to selinexor resulted in a strong shift in maximum efficacy on metastases (E_max_ ≈ 90%) compared to neuronal tissue (E_max_ ≈ 46%) (Figure 5D). Importantly, hCO viability remained high, despite potential compound accumulation in the tissue, demonstrating favorable selectivity against metastatic cancer cells. Caspase-3/7 was activated in a concentration-dependent manner, indicative of selinexor-induced apoptosis in A375-MBMs (Figure 5E) resulting in a concomitant reduction in Ki-67-positive metastases (Figure 5F). To determine if the metastatic regression correlated with nuclear export inhibition, we analyzed selinexor-treated A375-MBMs for a functional alteration of XPO1, the molecular target of selinexor. In the remaining cancer cells, treatment had induced a nuclear accumulation of TP53, a key XPO1 cargo,^51^ suggesting target engagement (Figure 5F). For a clinically relevant validation, we expanded the platform to patient-derived MelDCC lines. Over 7 days of co-culture with hCOs, we monitored a similar metastasis growth profile of MelDCC3- and MelDCC8-MBMs compared to A375-MBMs, although the overall fluorescence intensity differed (Figure 5G). Notably, colonized cancer cells were found in very close proximity to neuronal cells at the surface of each MBM model (Figure 5H). Additionally, we validated the MBM generation and assay workflow using male MelDCC8 cells and the sex-matched hiPSC line HHUUKDi009-A, reaching a similar assay window during colonization than the female models (A375-MBM based on hCOs derived from WISCi004-B or ZIPi013-B hiPSCs) or any of the mixed-sex models (Figures S4A and S4B). Following the previously optimized parameters for compound exposure, we then treated MBMs with 0.25 and 0.5 μM selinexor. Data revealed a concentration-dependent anti-metastatic activity in a clinically promising concentration range in both MBM models (Figure 5I). We observed a distinct sensitivity in A375- and MelDCC8-MBMs with a residual viability indicating a refractory subpopulation of cancer cells.

### Confirmation of selinexor’s BBB permeability

**Figure 6:**
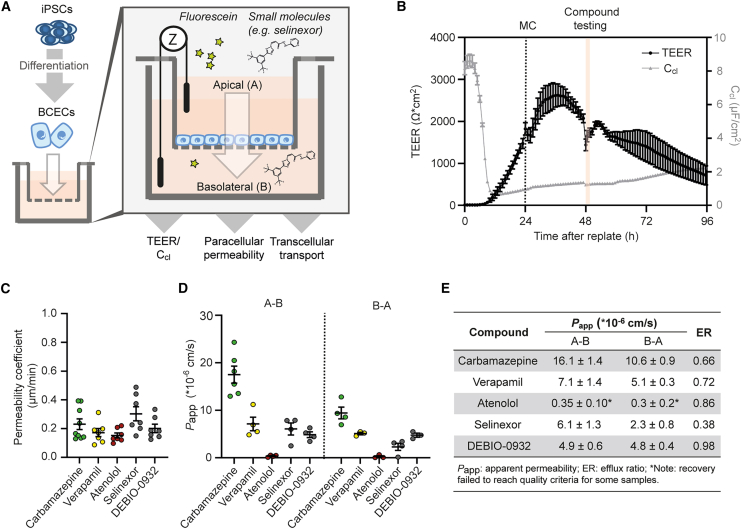
Blood-brain barrier permeation of selinexor and DEBIO-0932 tested in an hiPSC-derived brain capillary endothelial cell model (A) Schematic overview of small-molecule permeation and transport assay between apical (A) and basolateral (B) compartment using hiPSC-derived brain capillary endothelial cells (BCECs). Impedance (Z)-based measurements of transendothelial electrical resistance (TEER) and capacitance (C_cl_) as well as fluorescein permeability underlining blood-brain barrier (BBB) integrity. (B) Monitoring of TEER (left axis) and C_cl_ (right axis) to analyze BBB integrity over time after replating onto insert membranes (*n* = 3 independent differentiations). Data are presented as mean ± SEM. Labels indicate the time point of medium change (MC) and 1 h window for compound testing. (C) Analysis of fluorescein permeability coefficient during compound administration (*n* = 6–9 independent experiments). Data are presented as mean ± SEM. Carbamazepine (quick), verapamil (medium), and atenolol (slow permeation) serve as reference compounds. (D) Apparent permeability coefficient (*P*_app_) in both directions (apical to basolateral [A-B] and basolateral to apical [B-A]) after 1 h of selinexor or DEBIO-0932 exposure in comparison to reference compounds carbamazepine (quick), verapamil (medium), and atenolol (slow permeation) analyzed by mass spectrometry (*n* = 3–6 experiments). Data are presented as mean ± SEM. (E) Overview of calculated *P*_app_ values and efflux ratios (ERs) of selinexor, DEBIO-0932, and reference compounds.

Although the penetration of DCCs into the brain parenchyma may remodel the BBB into a blood-tumor barrier (BTB), it does not exclude the possibility of a selective barrier for systemically administered drugs.^54^ Since our hiPSC-derived MBM models do not incorporate aspects of BBB permeability, we tested the permeation of selinexor and DEBIO-0932 in a human iPSC-derived *in vitro* assay. We differentiated wild-type hiPSCs (WISCi004-B) into brain capillary endothelial cells (BCECs), as previously reported.^55^^,^^56^^,^^57^^,^^58^ To assess the transport of compounds across the BBB-like structure, we seeded BCECs differentiated for 8 days onto insert membranes (Figure 6A). We continuously measured transendothelial electrical resistance (TEER) values exceeding 1,000 Ω ∗cm^2^ over a 60-h period, along with capacitance values ranging from 1 to 2 μF/cm^2^, indicative of the formation of a tight BCEC monolayer^59^ (Figure 6B). We chose 48 h post-seeding as the treatment time point for 1 h exposure to 10 μM selinexor or DEBIO-0932. We assessed flux in both directions (apical to basolateral [A-B] and basolateral to apical [B-A]) and compared permeability values to reference compounds (carbamazepine [quick permeation], verapamil [medium permeation], and atenolol [slow permeation]). Sodium fluorescein solution was administered together with the test compounds and confirmed limited passive diffusion and maintenance of barrier integrity during the treatment period (Figure 6C). Mass spectrometry analysis revealed good permeability for both selinexor and DEBIO-0932, similar to Verapamil. We detected higher apparent permeability coefficients (*P*_app_) for selinexor in apical-to-basolateral permeation and relatively low efflux ratios, suggesting additional active uptake of these drugs from vessels to brain parenchyma, beyond passive permeation (Figures 6D and 6E). These data further indicate a good permeation with low efflux transport at the BBB, essential for achieving therapeutic selinexor concentrations and minimizing dosage requirements.

## Discussion

Improving the clinical management of BrM requires the availability of preclinical model systems that reproducibly capture key features of human disease, particularly in screening-compatible assay formats. It is already widely acknowledged that two-dimensional (melanoma) cell lines often fail to exhibit critical molecular and phenotypic alterations of metastatic cells and lack key structural and functional interactions with the metastatic niche.^60^ Since early metastatic colonization is the rate-limiting step in the metastatic cascade, adjuvant therapeutic targeting of the early metastatic seed, the DCCs, is key to prevent the onset of difficult to cure BrM. Our scalable and reproducible human MBM platform proposed here, combining hiPSC-derived hCOs with early metastatic cancer cells in a robust screening assay format, addresses a previously unmet need for improved preclinical studies. The model recapitulates key features of metastatic colonization, capturing the dynamic transition from DCCs to micrometastases within the brain TME. Notably, our model shows a strong metastasis-like signature, a pronounced EMT phenotype, and demonstrates intimate cancer cell interactions with both neuronal and astroglia-like cells, aligning with contemporary concepts of neurooncology.^61^

We have here demonstrated that we can profile anti-cancer drugs in screening mode across diverse melanoma cell types, including tumor-derived (A375) or patient-derived DCCs, representing varied melanoma differentiation phenotypes, genetic backgrounds, and biological sexes. Critically, the MBM platform’s utility extends beyond testing of therapeutics in early preclinical or clinical stages of drug development. It also provides a clinically relevant context to evaluate therapies for patients with established BrMs, addressing a key unmet need. Patients with BrM are often excluded from clinical trials, also due to the limited information on the ability of drugs to cross the BBB or BTB, hindering the development of new therapeutic opportunities targeting BrM.^8^ In addition to assessing sensitivity to targeted molecular therapies, this model also allows for the interrogation of unwanted side effects in the brain parenchyma, as shown here for the BBB-permeating XPO1 inhibitor selinexor. Our study aims to emphasize the differences in drug sensitivity of model systems, i.e., metastatic cancer cells in 2D or 3D, with or without niche tissue, and holds the potential to identify potent compounds that initiate MBM regression. The MBM platform may therefore provide a means to offer a relevant tissue context in which drugs, not previously tested for BrMs, could be easily evaluated and prioritized. This triaging strategy for metastasis drug screening, from less complex 2D models to a multicellular metastasis model system, could enable a rational, cost- and time-effective hit selection for further *in vivo* validation.

Prediction of treatment responses using *ex vivo* systems may pave the way to personalized medicine. Our proposed co-culture and drug screening workflow aligns with clinically relevant timelines, including postoperative recovery and histological analysis of biopsies. Given the regular availability of hCO and patient specimens, this platform could serve as an additional tool for exploring candidate therapies *ex vivo*, either as monotherapy or in combination with the standard of care. It could also support treatment decisions for individual (high-risk) BrM patients and/or certain subgroups. We implemented the colonization procedure with patient-derived specimens, which can also be potentially expanded to DCC lines from patients with different diagnoses, such as breast cancer. We are confident that the MBM platform will support optimal patient stratification and improve clinical trial design, thus promoting the impact of personalized medicine in oncology.

### Limitations of the study

We acknowledge that our approach to an experimental drug-screening platform targeting metastasis has limitations. The MBM model does not fully recapitulate the metastatic cascade due to missing vascularization of the hCOs. Therefore, we investigated compound permeability with an additional hiPSC-derived BBB model. The colonization in our model cannot fully recapitulate the *in vivo* situation due to a lack of peripheral immune cells and an incomplete ECM. Limits of the platform include availability of patient tissue, particularly on reporter-transduced DCC lines, which is a time-critical factor. All data shown here were derived from one melanoma cell line and three patients and may not fully represent the generalizability of the approach. Also, we cannot exclude any lack of sensitivity of DCC-derived MBMs due to prior exposure of patients to different treatment therapies.

## Resource availability

### Lead contact

Requests for further information and resources should be directed to and will be fulfilled by the lead contact, Dr. Ole Pless (ole.pless@itmp.fraunhofer.de).

### Materials availability

The A375-tdTomato cell line generated in this study will be made available on request but will require a completed materials transfer agreement. There are restrictions to the availability of MelDCC cell lines owing to hospital’s ethical regulations.

### Data and code availability

• mRNA sequencing data reported in this paper have been deposited at GEO: GSE293348 and are publicly available. Microscopy data reported in this paper will be shared by the lead contact upon request.
• This study does not report original code.
• Any additional information required to re-analyze the data reported in this study is available from the lead contact upon request.

## Acknowledgments

This work was supported by the 10.13039/501100001659German Research Foundation (Deutsche Forschungsgemeinschaft [DFG], SFB TRR 305, project number 429280966 [to K.E., M.W.-K., C.A.K., J.W., and O.P.]), by the Bundesministerium für Bildung und Forschung (BMBF) Clusters4Future project “BioDEL” to M.M. (03ZU1109GD) and O.P. (03ZU1109GB), by the Else-Kröner-Fresenius Foundation (2023_EKMS.03 to M.S.W.), by the Corona foundation (S0199/10110/2025 to M.S.W.), and by a short-term scientific mission grant from COST Action CA20140 “CorEuStem” (to K.K.). We thank the EMBL mesoscopic imaging facility Barcelona (head: Dr. James Swoger), the tissue engineering unit (head: Dr. Laura Batlle Morera) of the Centre for Genomic Regulation (CRG) Barcelona, and Kristin Hartmann (Experimental Pathology Core Facility, University Medical Center Hamburg-Eppendorf) for providing their support in sample preparation and image acquisition. In addition, we thank nanoAnalytics GmbH (Münster) and PreSens Precision Sensing GmbH (Regensburg) for resource and technical support and Norbert Garbow (Revvity) for support with the customized spheroid detection analysis. We thank Dr. Julian Kött and Dr. Falk Klose (University Medical Center Hamburg-Eppendorf, Department of Dermatology and Venerology) for critical reading and discussion of the manuscript. We are grateful to all patients for the donation of cancer cell specimen, which enabled the studies described here. Some figures were created with BioRender.

## Author contributions

Conceptualization, K.K. and O.P.; data curation, K.K., S.M.-R., T.N., F.-Z.R., U.H., A.Z., M.S.W., M.S., P.M., H.S., K.E., and S.K.; formal analysis, K.K., S.M.-R., T.N., F.-Z.R., P.K., U.H., A. Wittich, A.Z., M.S.W., M.W., M.S., J.H., and P.M.; funding acquisition, K.K., M.S.W., M.A.F., M.M., K.E., N.S.G., C.A.K., M.W.-K., J.W., and O.P.; investigation, K.K., S.M.-R., T.N., F.-Z.R., P.K., A. Weller, A. Wittich, M.S., J.H., P.M., D.M., H.S., and S.K.; methodology, K.K., S.M.-R., T.N., U.H., and J.W.; project administration, K.K., M.M., K.E., N.S.G., C.A.K., M.W.-K., J.W., and O.P.; resources, R.B., C.C., M.M., K.E., S.K., C.A.K., M.W.-K., J.W., and O.P.; software, A.Z. and M.S.W.; supervision, R.Z., R.B., M.A.F., M.M., K.E., N.S.G., C.A.K., M.W.-K., J.W., and O.P.; validation: K.K., S.M.-R., T.N., F.-Z.R., P.K., A. Weller, U.H., M.S., P.M., D.M., H.S., K.E., S.K., and M.W.-K.; visualization, K.K., U.H., M.S.W., M.W., M.S., H.S., K.E., and S.K.; writing – original draft, K.K. and O.P.; writing – review & editing, K.K., U.H., C.A.K., M.W.-K., J.W., and O.P. All co-authors contributed to the editing and discussion of the manuscript and approved the final version.

## Declaration of interests

The authors declare no competing interests.

## STAR★Methods

### Key resources table

**Table:** 

REAGENT or RESOURCE	SOURCE	IDENTIFIER
**Antibodies**		

Mouse monoclonal anti-MAP2 (Clone: M13)	Invitrogen	Cat#13–1500; RRID: AB_2533001
Rabbit polyclonal anti-PAX6	Abcam	Cat# ab5790; RRID: AB_305110
Rabbit polyclonal anti-SATB2	Abcam	Cat# ab34735; RRID: AB_2301417
Rabbit polyclonal anti-RFP	Antibodies-Online	Cat# ABIN129578; RRID: AB_10781500
Mouse monoclonal anti-RFP	Invitrogen	Cat# MA5-15257; RRID: AB_10999796
Chicken polyclonal anti-RFP (mScarlet)	Synaptic Systems	Cat# 409 006; RRID: AB_2725776
Mouse monoclonal anti-Fibronectin (Clone: 1G10F9)	Proteintech	Cat# 66042-1-Ig; RRID: AB_11182385
Rabbit polyclonal anti-COL8A1	Proteintech	Cat# 17251-1-AP; RRID: AB_10696173
Mouse monoclonal anti-Vimentin	Novocastra	Cat# NCL-L-VIM-V9; RRID: AB_564055
Rabbit monoclonal anti-GFAP	Synaptic Systems	Cat# 173 008; RRID: AB_2864794
Rabbit monoclonal anti-GFAP	Synaptic Systems	Cat# 173 208; RRID: AB_2864795
Mouse monoclonal anti-p53 (Clone: DO-1)	Invitrogen	Cat# MA5-12571; RRID: AB_10986581
Rabbit polyclonal anti-Ki-67	Abcam	Cat# ab15580; RRID: AB_443209
Goat anti-rabbit AF635	Invitrogen	Cat# A-31576; RRID: AB_10374303
Donkey anti-rabbit AF488	Invitrogen	Cat# A-21206; RRID: AB_2535792
Goat anti-mouse AF633	Invitrogen	Cat# A-21050; RRID: AB_2535718
Goat anti-mouse AF488	Invitrogen	Cat# A-11001; RRID: AB_2534069
Goat anti-chicken AF488	Invitrogen	Cat# A-11039; RRID: AB_2534096
Mouse anti-CD271 (NGFR) Pacific Blue	BioLegend	Cat# 345131; RRID: AB_2894480
Mouse anti-Melan-A AF647	Santa Cruz Biotechnology	Cat# sc-20032 AF647; RRID: AB_3106925
Pacific Blue mouse IgG1, κ isotype control	BioLegend	Cat# 400131; RRID: AB_2923473
Alexa Fluor 647 mouse IgG1, κ isotype control	BioLegend	Cat# 400155; RRID: AB_2832978

**Chemicals, peptides, and recombinant proteins**		

Gentle Cell Dissociation Reagent	STEMCELL Technologies	Cat# 100-0485
Matrigel	Corning	Cat# 356231
Y-27632 (Dihydrochloride)	STEMCELL Technologies	Cat# 72304
Anti-adherence rinsing solution	STEMCELL Technologies	Cat# 07010
Penicillin-streptomycin	Capricorn Scientific	Cat# PS-B
Penicillin-streptomycin	PAN-Biotech	Cat# P06-07100
DMEM/F-12	Gibco	Cat# 21331046
KnockOut serum replacement	Gibco	Cat# 10828028
L-glutamine	Capricorn Scientific	Cat# GLN-B
MEM Non-Essential Amino Acids Solution	Gibco	Cat# 11140050
β-Mercaptoethanol	Gibco	Cat# 31350010
Human Endothelial Serum-Free Medium	Gibco	Cat# 11111044
B-27	Gibco	Cat# 17504044
FGF	PeproTech	Cat# 100-18b
Retinoic acid	STEMCELL Technologies	Cat# 72262
Collagen type IV	Sigma-Aldrich	Cat# C5533
Fibronectin	Gibco	Cat# 33016015
RPMI 1640	PAN-Biotech	Cat# P04-17500
FBS good	PAN-Biotech	Cat# P40-37500
FBS advanced	Capricorn Scientific	Cat# 10-FBS-11F
GlutaMAX Supplement	Thermo Fisher Scientific	Cat# 35050038
TrypLE Express Enzyme	Gibco	Cat# 12604013
Trypsin-EDTA	PAN-Biotech	Cat# P10-024100
Puromycin-dihydrochloride	Gibco	Cat# A1113803
Geltrex	Gibco	Cat# A1413202
Formaldehyde	Carl Roth	Cat# 6742.5
Tween 20	Sigma-Aldrich	Cat# P7949
Methanol	VWR International	Cat# 20846.292
Dimethyl sulfoxide (DMSO)	Sigma-Aldrich	Cat# D5879
Hydrogen peroxide solution	Sigma-Aldrich	Cat# H1009
Triton X-100	Carl Roth	Cat# 3051.3
Low melt agarose	Bio-Rad Laboratories	Cat# 1613111
Benzyl 4-hydroxybenzoate	Sigma-Aldrich	Cat# 07389
Benzyl alcohol	Sigma-Aldrich	Cat# 108006
Poly-L-Ornithine	Sigma-Aldrich	Cat# P4957
Laminin	Sigma-Aldrich	Cat# L2020
Universal R buffer	Aptum Biologics	Cat# AP0530-500
Bovines Serum Albumin (BSA)	Carl Roth	Cat# 8076.4
Hoechst 33342	Invitrogen	Cat# H3570
TrueBlack	Biotium	Cat# 23007
ProLong Diamond Antifade Mounting Medium	Invitrogen	Cat# P36965
Hematoxylin	Ventana Roche	Cat# 760-2021
Bluing Reagent	Ventana Roche	Cat# 760-2037
qPCR Master Mix	Applied Biosystems	Cat# 4369016
Sodium chloride	Carl Roth	Cat# 0601
Potassium chloride	Sigma-Aldrich	Cat# P9541
Magnesium chloride	Carl Roth	Cat# KK36
Calcium chloride	Sigma-Aldrich	Cat# C1016
HEPES	Sigma-Aldrich	Cat# H3375
D-glucose	Sigma-Aldrich	Cat# X997
Potassium D-gluconate	Sigma-Aldrich	Cat# G4500
EGTA	Carl Roth	Cat# 3054
ATP magnesium	Sigma-Aldrich	Cat# A9187
Formaldehyde	Sigma-Aldrich	Cat# 252549
Glutaraldehyde	EMS	Cat# 16400
Paraformaldehyde	EMS	Cat# 19200
Cacodylic Acid-Sodium Salt	EMS	Cat# 12310
Sucrose	EMS	Cat# 21600
Osmium tetroxide	EMS	Cat# 19110
Ethanol	Sigma-Aldrich	Cat# 32221-M
Embed 812	EMS	Cat# 14900
D.D.S.A.	EMS	Cat# 13710
NMA	EMS	Cat# 19000
DMP-30	EMS	Cat# 13600
Uranyl acetate	Fluka	Cat# 94260
Ultrostain 2	Leica Biosystems	Cat# 16707235
Dulbecco's Phosphate Buffered Saline (D-PBS)	Sigma-Aldrich	Cat# D8537
Ethanol	VWR International	Cat# 85651
BD Cytofix/Cytoperm Fixation and Permeabilization Solution	BD Biosciences	Cat# 554722
FluoroFix Buffer	BioLegend	Cat# 422101
Intracellular Staining Permeabilization Wash Buffer	BioLegend	Cat# 421002
Anti-cancer approved drug library	Hölzel Diagnostika Handels GmbH	Cat# L2110-1-TM
Temsirolimus	Biomol	Cat# TGM-T2145
GSK343	Biomol	Cat# TGM-T6059
Bortezomib	Hölzel Diagnostika Handels GmbH	Cat# TMO-T2399
GrowDex-T	Revvity	Cat# 200103002
Selinexor	Selleckchem	Cat# S7252
DEBIO-0932	MedChemExpress	Cat# HY-13469
Carbamazepine	Sigma-Aldrich	Cat# C4024
Atenolol	Sigma-Aldrich	Cat# A7655
Verapamil hydrochloride	Sigma-Aldrich	Cat# V4629
Fluorescein sodium salt	Sigma-Aldrich	Cat# F6377
Carbutamid	AbbVie	In-house synthesis
Water with 0.1% (v/v) formic acid	VWR International	Cat# 84867.290
Acetonitrile	VWR International	Cat# 83640.290
Ammonium bicarbonate	Sigma-Aldrich	Cat# 5.33005
Ammonium hydroxide	Honeywell International	Cat# 30501-1L

**Critical commercial kits**		

mTeSR Plus	STEMCELL Technologies	Cat# 100-0276
Venor GeM Sample Preparation Kit	Minerva Biolabs	Cat# 56-1050
Venor GeM OneStep Kit	Minerva Biolabs	Cat# 11-8100
STEMdiff Dorsal Forebrain Organoid Differentiation Kit	STEMCELL Technologies	Cat# 08620
STEMdiff Neural Organoid Maintenance Kit	STEMCELL Technologies	Cat# 100-0120
UltraView Universal DAB Detection Kit	Roche	Cat# 760-500
QIAshredder	QIAGEN	Cat# 79656
RNeasy Micro Kit	QIAGEN	Cat# 74004
RevertAid H Minus First Strand cDNA Synthesis Kit	Thermo Fisher Scientific	Cat# K1632
TaqMan Primer Assay TBP	Thermo Fisher Scientific	TaqMan Assay ID: Hs00427620_m1
TaqMan Primer Assay MAP2	Thermo Fisher Scientific	TaqMan Assay ID: Hs00258900_m1
TaqMan Primer Assay PAX6	Thermo Fisher Scientific	TaqMan Assay ID: Hs01088114_m1
TaqMan Primer Assay VGLUT1	Thermo Fisher Scientific	TaqMan Assay ID: Hs00220404_m1
TaqMan Primer Assay BSN	Thermo Fisher Scientific	TaqMan Assay ID: Hs01109152_m1
SYLGARD 184 Silicone Elastomer Kit	Dow	N/A
ATPlite 1step Luminescence Assay System	Revvity	Cat# 6016736
Caspase-Glo® 3/7 3D Assay	Promega	Cat# G8981
CellTiter-Glo 3D Cell Viability Assay	Promega	Cat# G9682
Human CXCL10/IP-10 ELISA Kit - Quantikine	Bio-Techne	Cat# DIP100

**Deposited data**		

Bulk RNA seq dataset	This paper	GEO: GSE293348

**Experimental models: Cell lines**		

Human iPSC line: WISCi004-B	WiCell Research Institute	RRID: CVCL_C437
Human iPSC line: ZIPi013-B	ZIP gGmbH; Tandon et al.^62^	RRID: CVCL_UF44
Human iPSC line: HHUUKDi009-A	HHU Düsseldorf; Lorenz et al.^63^	RRID: CVCL_B3T9
Human melanoma cell line: A375	ATCC	Cat# CRL-1619; RRID: CVCL_0132
Human melanoma cell line: MelDCC3	University of Regensburg/Fraunhofer ITEM-R	N/A
Human melanoma cell line: MelDCC8	University of Regensburg/Fraunhofer ITEM-R	N/A
Human melanoma cell line: MelDCC11	University of Regensburg/Fraunhofer ITEM-R	N/A

**Recombinant DNA**		

pRRL-CMV-GFP-puro	Swiss Institute of Cancer Research	N/A
LeGO-T2	University Medical Center Hamburg-Eppendorf	Addgene, Cat#27342; RRID: Addgene_27342

**Software and algorithm**		

Kaleido software	Revvity	N/A
VisiSens scientifical software	PreSens	https://www.presens.de/de/produkte/detail/visisens-scientifical-software
ImageJ 1.54	NIH	https://imagej.net; RRID: SCR_003070
Imaris 10.0.0	Oxford Instruments	http://www.bitplane.com/imaris/imaris; RRID: SCR_007370
Harmony	Revvity	https://www.perkinelmer.com/product/harmony-5-1-office-hh17000012; RRID: SCR_023543
Patch Master V2x80	HEKA Elektronik	https://www.heka.com/downloads/downloads_main.html#down_patchmaster
Origin Pro	OriginLab	http://www.originlab.com/index.aspx?go=PRODUCTS/Origin; RRID: SCR_014212
STAR v.2.4	Dobin et al.^64^	http://code.google.com/p/rna-star/; RRID: SCR_004463
featureCounts v.1.5.1	Liao et al.^65^	http://bioinf.wehi.edu.au/featureCounts/; RRID: SCR_012919
DESeq2 v.3.12	Love et al.^66^	https://bioconductor.org/packages/release/bioc/html/DESeq2.html; RRID: SCR_015687
biomaRt v.4.0	Durinck et al.^67^	https://bioconductor.org/packages/biomaRt/; RRID: SCR_019214
clusterProfiler	Yu et al.^68^	http://bioconductor.org/packages/release/bioc/html/clusterProfiler.html; RRID: SCR_016884
STRING	Global Biodata Coalition and ELIXIR	http://string.embl.de/; RRID: SCR_005223
FlowJo	BD Biosciences	https://www.flowjo.com/solutions/flowjo; RRID: SCR_008520
Analyst 1.7.2	Sciex	https://sciex.com/products/software/analyst-software
GraphPad Prism 10	GraphPad	http://www.graphpad.com/; RRID: SCR_002798

**Other**		

6-well plates	Greiner Bio-One	Cat# 657-160
AggreWell 800	STEMCELL Technologies	Cat# 34815
96-well ultra-low attachment plates	Corning	Cat# 7007
Nunc 6-well plates	Thermo Scientific	Cat# 140675
ThinCert cell culture inserts	Greiner Bio-One	Cat# 662-641
Corning mini bioreactor	Sigma-Aldrich	Cat# CLS431720
24-well plates	Greiner Bio-One	Cat# 662160
35 mm Petri dish	Thermo Scientific	Cat# 130180
Echo Qualified 384-well plates	Labcyte	Cat# 001-16128
384-well plates	Greiner Bio-One	Cat# 781091
96-well cell culture microplate, CELLSTAR, black	Greiner Bio-One	Cat# 655090
96-well cell culture microplate, CELLSTAR, white	Greiner Bio-One	Cat# 655083
Breathseal sealer	Greiner Bio-One	Cat# 676050
Multi-channel micropipettes	Brand	N/A
Cell culture warming plate	Labotect	N/A
EnSight multimode plate reader	Revvity	N/A
Oxygen-sensor foils	PreSens	Cat# SF-RPSu4
VisiSens TD	PreSens	N/A
CS-Mount Lens	ArduCam	Cat# CS2325ZM01
Silicon glue	RS Components	N/A
Muvi SPIM	Bruker	N/A
ASP300S tissue processor	Leica biosystems	N/A
Antigen Retriever 2100	Aptum	N/A
Operetta CLS High Content Imaging System	Revvity	N/A
Ventana BenchMark XT autostainer	Ventana	N/A
Zeiss Axioscope 5 microscope	Zeiss	N/A
Axiocam 208 color camera	Zeiss	N/A
NanoDrop 1000 spectrophotometer	Thermo Fisher Scientific	N/A
QuantStudio 6 Flex Real-Time-PCR-System	Applied Biosystems	N/A
EPC 10 USB System with Red Star Headstage	Warner Instruments	Cat# 89-5273
Eclipse FN1 microscope	Nikon	N/A
LYNX microscopy tissue processor	Reichert-Jung	N/A
Side-mounted 2kx2k camera	TRS TRÖNDLE Restlichtverstärkersysteme	N/A
LEO 912AB electron-microscope	ZEISS	N/A
Ultracut S Microtome	Reichert-Jung	N/A
Diamond knives	DiATOME	N/A
Autosamdri-815	Tousimi	N/A
Goldpalladium	Leica Microsystems	Cat# EM ACE 200
Crossbeam 550	Zeiss	N/A
FACSymphony A3	BD Biosciences	N/A
Gallios 10C 3L Flow Cytometer	Beckmann Coulter	N/A
Echo 550 liquid handler	Labcyte	N/A
MultiDrop	Thermo Fisher Scientific	N/A
Standard Tube Cassettes	Steinle Labtechnology	N/A
EnVision Multimode Plate Reader	Revvity	N/A
cellZscope	nanoAnalytics	N/A
Infinite M1000 Pro	TECAN	N/A
MTS4 microtiter shaker	IKA	N/A
Acquity UPLC	Waters	N/A
Sciex 6500+	Sciex	N/A
BEH C18 column	Waters	Cat# 186002349

### Experimental model and study participant details

All experiments involving cells originated from patient biopsies and were approved by the medical ethics committee of the University of Regensburg. MelDCC lines were derived from lymph node biopsies of melanoma patients after tumor resection and were propagated directly *in vitro* or in xenografts as described previously^16^ and S.M.-R., M.W.-K., C.A.K., K. Weidele, C. Werno, and S. Treitschke (unpublished data).

### Method details

#### Cultivation of human hiPSCs

Human wild-type iPSCs (WISCi004-B, WiCell), ZIPi013-B,^62^ and HHUUKDi009-A^63^ were maintained on Matrigel (Corning, 356231)-coated 6-well plates (Greiner Bio-One, 657-160) in mTeSR Plus medium (STEMCELL Technologies, 100–0276). Passaging was performed approximately every five days at 50–70% confluency, using Gentle Cell Dissociation Reagent (STEMCELL Technologies, 100–0485), and medium was refreshed every other day. To routinely confirm the absence of mycoplasma contamination, the Venor GeM Sample Preparation Kit (Minerva Biolabs, 56–1050) and Venor GeM OneStep Kit (Minerva Biolabs, 11–8100) were utilized according to the manufacturer’s instructions. All cell cultures were conducted at 37°C and 5% CO_2_ in humidified incubators, unless otherwise stated.

#### Differentiation of hiPSC-derived cortical organoids

Differentiation of hiPSCs into hCO was performed as previously described^27^^,^^31^ using the STEMdiff Dorsal Forebrain Organoid Differentiation Kit (STEMCELL Technologies, 08620) with some adaptations. First, hiPSCs at a confluency of 60–80% were detached using Gentle Cell Dissociation Reagent (STEMCELL Technologies, 100–0485) and 3 × 10^6^ cells transferred to each well of an AggreWell 800 microwell culture plate (STEMCELL Technologies, 34815) in forebrain organoid formation medium supplemented with 10 μM Y-27632 (STEMCELL Technologies, 72304). The medium was changed daily using forebrain organoid formation medium without Y-27632. On day 6, embryoid bodies were individually transferred to 96-well clear round bottom ultra-low attachment microplates (Corning, 7007) on a cell culture warming plate (Labotect) using sterilized wide-bore tips (prepared in-house) coated with anti-adherence rinsing solution (STEMCELL Technologies, 07010). To prevent evaporation and minimize plate effects, border wells were filled with D-PBS. Until day 25, hCOs were cultured in organoid expansion medium (200 μL/well), followed by organoid differentiation medium (200 μL/well). From day 43, hCOs were maintained in organoid maintenance medium (200 μL/well) using the STEMdiff Neural Organoid Maintenance Kit (STEMCELL Technologies, 100–0120). All medium changes were carefully performed as partial medium changes (50–75% of the volume) using multi-channel micropipettes (Brand) three times a week. Organoid media used in microplates were supplemented with 0.5% penicillin-streptomycin (Capricorn Scientific, PS-B). Each batch contained a maximum of 240 hCOs distributed across four 96-well plates.

#### Differentiation of hiPSC-derived BCECs for *in vitro* BBB models

The brain capillary endothelial cell (BCEC) differentiation was performed as reported previously^56^^,^^69^. Differentiation of hiPSCs (WISCi004-B), cultured in mTeSR Plus (STEMCELL Technologies, 100–0276) on Matrigel (Corning, 356231)-coated Nunc 6-well plates (Thermo Scientific, 140675), was initiated three days post-seeding. At an optimal cell density of 2–4 × 10^3^ cells/cm^2^, the medium was changed to unconditioned medium containing 78.5% DMEM/F-12 (without Glutamine; Gibco, 21331046), 20% KnockOut serum replacement (Gibco, 10828028), 1 mM L-glutamine (Capricorn Scientific, GLN-B), 1% non-essential amino acids (Gibco, 11140050), and 0.1 mM β-mercaptoethanol (Gibco, 31350010). At day 6, medium was switched to endothelial cell (EC) medium containing 99.5% Human Endothelial Serum-Free Medium (Gibco, 11111044) and 0.5% B-27 (Gibco, 17504044), supplemented with 20 ng/mL hFGF (PeproTech, 100-18b) and 10 μM retinoic acid (RA) (STEMCELL Technologies, 72262). By day 8 of differentiation, 1 × 10^6^ cells/cm^2^ were seeded onto Collagen IV (Sigma-Aldrich, C5533)/Fibronectin (Gibco, 33016015)-coated inserts (0.4 μm pore size, transparent, 24-well format; Greiner Bio-One, 662-641). The next day, BCECs were adapted to EC medium without hFGF and RA. We monitored transendothelial electrical resistance (TEER) to assess the integrity of the established *in vitro* BBB, including only inserts with values ≥1000 Ω∗cm^2^ for subsequent permeability studies.

#### 2D cultivation of melanoma cells

MelDCC lines were derived from DCC xenografts or directly propagated *in vitro* as described previously^16^ and S.M.-R., M.W.-K., C.A.K., K. Weidele, C. Werno, and S. Treitschke (unpublished data). Patient origin was verified by STR analysis (Cell-ID, Promega), melanoma origin by a surgical pathologist, and genotype by copy number alterations. MelDCCs were cultured in complete MelDCC medium containing RPMI 1640 (without L-Glutamine, with 2.0 g/L NaHCO_3_; PAN-Biotech, P04-17500) with 10% FBS good (PAN-Biotech, P40-37500), 1% penicillin-streptomycin (PAN-Biotech, P06-07100), and 1% GlutaMAX Supplement (Thermo Fisher Scientific, 35050038). Depending on the growth rate, cells were passaged every three to five days using TrypLE Express Enzyme (Gibco, 12604013) in ratios between 1:2 and 1:10. A375 (CRL-1619) cell line was obtained from ATCC. Cells were maintained in complete A375 medium containing DMEM/F-12 (without Glutamine; Thermo Fisher Scientific, 21331046) with 10% FBS advanced (Capricorn Scientific, 10-FBS-11F), 1% penicillin-streptomycin (Capricorn Scientific, PS-B) and 2 mM L-Glutamine (Capricorn Scientific, GLN-B) and regularly passaged every three to four days using 0.05% Trypsin/0.02% EDTA (PAN Biotech, P10-024100). All cells were regularly tested for mycoplasma contamination.

#### Lentiviral transduction of melanoma cells

To induce and restrict fluorescence reporter protein expression to cancer cells only, we produced stable lines prior to co-cultivation. MelDCCs were transduced using the lentiviral vector pRRL-CMV-GFP-puro (kindly provided by Stephan Duss, ISREC, Swiss Institute of Cancer Research, Lausanne). A375-tdTomato reporter cell line was generated by lentiviral transduction using the LeGO-T2-Puro vector (kindly provided by Kristoffer Riecken, University Medical Center Hamburg-Eppendorf). Antibiotic selection was performed using puromycin-dihydrochloride (Gibco, A1113803), and tdTomato and GFP expressing cells were cryopreserved for follow-up experiments.

#### Melanoma brain metastases models

43-day-old hCOs with visible integrity were co-cultured with fluorescence-labeled melanoma cells for seven days. Therefore, melanoma cells from 2D maintenance cultures were detached as described before at a confluency of 60–90% and 1 × 10^4^ cells were applied by performing a regular medium change to each hCO followed by centrifugation for 3 min at 100 g. Co-culture formation was done at 37°C for 48 h in organoid maintenance medium. All medium changes were carried out with organoid maintenance medium supplemented with 0.5% penicillin-streptomycin (Capricorn Scientific, PS-B). They were carefully performed as partial medium changes (50–75% of the volume) using multi-channel micropipettes (Brand) three times a week. At day 50 (day 43 + 7), MBM models were used for downstream analysis as well as efficacy and safety studies.

#### MelDCC tumoroid propagation using semi-solid culture

Tumoroids were grown in Corning mini bioreactor centrifuge tubes with vented caps (Sigma-Aldrich, CLS431720). 3.5 × 10^5^ single cells from 2D cultures were transferred into each bioreactor and cultured in 5 mL semi-solid culture medium containing complete MelDCC medium supplemented with 5% Geltrex LDEV-Free Reduced Growth Factor Basement Membrane Matrix (Gibco, A1413202). Every three to four days, 2.5 mL of the semi-solid culture medium was added, and the suspension was mixed by pipetting up and down approximately 10 times using a 1 mL pipette tip. Tumoroids were harvested on day 10 for drug screening.

#### Live imaging

Brightfield and fluorescence imaging of hCOs and MBMs at different stages were conducted using the EnSight multimode plate reader (Revvity). Live imaging allowed monitoring of morphological parameters (organoid area and roundness) as well as the fluorescence intensity of MBMs with or without compound exposure, and did not exceed 15 min imaging time per plate. Single images were processed as z-stacks (seven images spanning 1800 μm) for a representative image and plate overviews. Images were analyzed by spheroid detection using Kaleido software (Revvity) with object detection settings (tuning of brightness limit: 0.6, flatfield correction scale of brightfield: 0, minimum object size: 100,000 μm^2^, minimum contrast requirements: very relaxed, ring-shaped objects: yes, largest objective, only: yes) and well detection settings (channel: automatic, exclude well margin 400 px, mode: small well, well dimensions: automatic, well shape: round, well diameter: 6.3 mm). Analyzed fluorescence data exclusively accounted for the fluorescence intensity of cancer cells within the detected organoid region. For MBM values, the fluorescence background of hCOs in organoid maintenance medium was subtracted.

#### Oxygen measurements

Oxygen levels were monitored using VisiSens TD (PreSens) at 37°C and 5% CO_2_ in a dry incubator. VisiSens TD was customized with a CS-Mount Lens (CS2325ZM01, ArduCam), providing the necessary field of view and magnification to monitor the organoids with sufficient lateral resolution. The oxygen-sensitive sensor foil was glued to the bottom of a standard 24- well cell-culture plate (Greiner Bio-One, 662160) using a biocompatible silicon glue (RS Components). Before use, the sensor plates were treated with argon plasma for 1 min hCOs at day 43 were transferred to Poly-L-Ornithine/Laminin (Sigma-Aldrich, P4957, L2020) coated oxygen-sensor foils (PreSens, SF-RPSu4) and cultured in organoid maintenance medium (STEMCELL Technologies). Oxygen imaging was started directly after organoid transfer. An image was taken every 5 min over the time course of 12 h. Data was analyzed using the VisiSens Scientifical software (PreSens) and ImageJ 1.54 (NIH). Sensors were calibrated via a 2-point calibration at 0% and 100% air saturation. A solution of sodium sulfite in medium served as 0% calibration solution.

#### Whole-mount imaging

Staining, clearing, and imaging procedures were based on previous reports.^70^ A375-MBMs were transferred to a 4% formaldehyde (Carl Roth, 6742.5) solution for 24 h. Samples were then incubated in 0.1% Tween 20 (Sigma-Aldrich, P7949) in D-PBS, followed by methanol (VWR, 20846.292) dehydration series, each 20 min. Samples can be stored in 100% methanol at −20°C. Incubation in methanol/DMSO/H_2_O_2_ solution (VWR International, 20846.292; Sigma-Aldrich, D5879; Sigma-Aldrich, H1009) in a ratio of 2:1:3 was conducted at 4°C overnight, followed by a methanol rehydration series, each for 20 min, and washing in 0.1% Tween 20 in D-PBS. Permeabilization and blocking were subsequently achieved using 0.1% Triton X-100 (Carl Roth, 3051.3) and 10% BSA (Carl Roth, 8076.4) in D-PBS overnight. Primary antibodies MAP2 (Invitrogen, 13–1500) and RFP (Antibodies-Online, ABIN129578) were incubated in a dilution of 1:100 in permeabilization and blocking solution supplemented with 5% DMSO (Sigma-Aldrich, D5879) on an orbital shaker for three days at room temperature. After washing in 0.1% Triton X-100 in D-PBS for 1 day, samples were incubated in secondary antibodies listed in the key resources table 1:500 for three days, washed, and embedded in 1% low melt agarose (Bio-Rad Laboratories, 1613111). Embedded samples were incubated in 100% methanol followed by optical clearing in BABB solution (1:2 Benzylalcohol:Benyzlbenzoate; Sigma-Aldrich) for 24 h each. Samples were imaged using the light-sheet microscope Muvi SPIM (Bruker) with a 10× objective in BABB clearing solution. 3D data stacks were reconstructed using Imaris software 10.0.0 (Oxford Instruments). Alpha blending was applied to the images to adjust transparency and improve detail perception.

#### Immunohistochemistry

2D cell cultures of A375 cells were fixed in a 4% formaldehyde solution for 20 min, scratched, and pelleted before paraffin embedding. Whole hCOs and A375-MBMs were fixed in 4% formaldehyde for 24 h, protected in 3% agarose prior to embedding, dehydrated using an ASP300S tissue processor (Leica Biosystems), and embedded in paraffin. Sections were cut at 3 μm, followed by deparaffination, rehydration, and antigen retrieval by pressure boiling in Universal R buffer (Aptum Biologics, AP0530-500) for 20 min using an Antigen Retriever (Aptum, 2100). For histological assessment, sections were stained with hematoxylin and eosin (H&E) according to standard procedures. For immunohistochemical staining, sections were permeabilized in 0.2% Triton X-100 (Carl Roth, 2051), blocked in 3% BSA (Carl Roth, 8076.4) and 0.1% Tween 20 (Sigma-Aldrich, P7949) in D-PBS and primary antibodies MAP2 (1:500, Invitrogen, 13–1500), PAX6 (1:200, Abcam, ab5790), SATB2 (1:500, Abcam, ab34735), RFP (1:1000, Antibodies-Online, ABIN129578), RFP (1:500, Invitrogen, MA5-15257), RFP (1:100, Synaptic Systems, 409006), Fibronectin (1:300, Proteintech, 66042-1-Ig), COL8A1 (1:50, Proteintech, 17251-1-AP), GFAP (1:2000, Synaptic Systems, 173008 and 1:200, Synaptic Systems, 173208), Ki-67 (1:500, Abcam, ab15580) and p53 (1:50, Invitrogen, MA5-12571) incubated over night at 4°C. The next day, sections were thoroughly washed in 0.1% Tween 20 in D-PBS and cold tap water, incubated with secondary antibodies listed in the key resources table in a dilution of 1:500 for 1 h, and thoroughly washed again. Nuclei were stained using 1 μg/μL Hoechst 33342 (Invitrogen, H3570). TrueBlack (Biotium, 23007) was shortly applied to mask autofluorescence before sections were mounted in ProLong. Diamond Antifade Mounting Medium (Invitrogen, P36965) with glass coverslips. We used the Operetta CLS High Content Imaging System (Revvity) with a 40× objective and confocal mode to image tissue sections on slide holders. Images covering full samples were acquired with 20% overlap and reconstructed using the Harmony software (Revvity). For comparative analysis of immunostaining, the samples were processed in parallel and imaged using the same settings.

GFAP-DAB and Vimentin (1:750, Novocastra, NCL-L-VIM-V9) staining was performed using the Ventana BenchMark XT autostainer (Ventana/Roche). After dewaxing and inactivation of endogenous peroxidases (3% hydrogen peroxide), sections were blocked and afterward incubated with the primary antibody (see above). For detection of specific binding and DAB staining, the UltraView Universal DAB Detection Kit (Roche, 760-500) was used, which contains both anti-mouse and anti-rabbit secondary antibodies. Counterstaining and bluing were performed with Hematoxylin (Ventana/Roche, 760–2021) and Bluing Reagent (Ventana/Roche, 760–2037) for 4 min. Subsequently, stained sections were mounted in mounting medium. Representative pictures were taken with a Zeiss Axioscope 5 microscope (Zeiss) and an Axiocam 208 color camera (Zeiss).

#### Quantitative reverse-transcription PCR analysis

Total RNA was extracted from pooled hCOs (*n* = 3 per batch) and corresponding hiPSC cultures using QIAshredder (QIAGEN, 79656) and the RNeasy Micro Kit (QIAGEN, 74004). RNA concentration was measured by NanoDrop 1000 spectrophotometer (Thermo Fisher Scientific), followed by cDNA synthesis using RevertAid H Minus First Strand cDNA Synthesis Kit (Thermo Fisher Scientific, K1632). cDNA synthesis was carried out using the following protocol: 5 min at 25°C, 60 min at 42°C, and 5 min at 70°C, followed by cooling to 4°C. cDNA was diluted to reach a 10 ng/μL concentration for the PCR reaction. Gene expression analysis for *TBP*, *MAP2*, *PAX6*, *VGLUT1*, and *BSN* was performed using qPCR Master Mix (Applied Biosystems, 4369016), with TaqMan primers listed in the key resources table. We used the QuantStudio 6 Flex Real-Time-PCR-System (Applied Biosystems) with the following protocol: 2 min at 50°C, 10 min at 95°C, 15 s at 95°C (for 40 cycles), and 1 min at 60°C. All measurements were performed in triplicate. hCO CT values were analyzed using the 2^−ΔΔCT^ method, normalized to the reference gene TBP and to the corresponding hiPSC control, and reported as Log2 fold change.

#### Patch clamp analysis

50-day-old hCOs were seeded on Poly-L-Ornithine/Laminin (Sigma-Aldrich, P4957, L2020) coated glass coverslips in organoid maintenance medium supplemented with 10 μM Y-27632 (STEMCELL Technologies, 72304). The next day, the medium was replaced with fresh organoid maintenance medium. Electrophysiology measurements were performed two to three days post-seeding as previously described with a few modifications.^71^ Coverslips were transferred to a 35 mm Petri dish (Thermo Scientific, 130180), washed three times and submerged in bath solution (140 mM NaCl (Carl Roth, 0601), 2.4 mM KCl (Sigma-Aldrich, P9541), 1.3 mM MgCl_2_ (Carl Roth, KK36), 2.5 mM CaCl_2_ (Sigma-Aldrich, C1016), 10 mM HEPES (Sigma-Aldrich, H3375), 10 mM D-glucose (Sigma-Aldrich, X997), pH 7.4) at room temperature. Pipettes were filled with pipette solution consisting of 125 mM potassium gluconate (Sigma-Aldrich, G4500), 10 mM NaCl (Carl Roth,0601), 1 mM EGTA (Carl Roth, 3054), 4 mM magnesium ATP (Sigma-Aldrich, A9187), 10 mM HEPES (Sigma-Aldrich, H3375), 10 mM D-glucose (Sigma-Aldrich, X997), pH 7.4, at room temperature. For data recording, a HEKA EPC 10 USB patch-clamp amplifier with a Red Star Headstage (Warner Instruments, 89–5273) and the Patch Master V2x80 software (HEKA Elektronik) were employed. A Bessel low-pass filter was set to 2.9 kHz, and capacitance and series resistance were automatically compensated. The set-up was mounted to an upright Nikon Eclipse FN1 microscope with a Nikon CFI TU Plan EPI ELWD 50x N.A. 0.60/W.D. 11.00 mm objective.

Outgrowing neurons from organoids on coverslips were patched, and three batches were analyzed. Their electrophysiology was assessed by their response to a current step function in current clamp mode from −5 to 40 pA in 5 pA steps. Currents were applied for 600 ms. Solely, neurons exhibiting action potential (AP) trains (n_AP_ ≥ 3) were further analyzed, i.e., *n* = 8 cells per batch. For AP characterization, the first AP of an AP train at rheobase current was examined. AP threshold was defined as the membrane potential where the second derivative became different from zero, AP height as the threshold to peak, and AP duration as the full-width half-maximum of the AP. The resting membrane potential was determined in current-clamp mode at a current set to zero, the membrane capacitance by the Patch Master software. Inward and outward currents were recorded in whole-cell mode by a voltage step function from −70 to +30 mV in 10 mV steps. Currents were applied for 100 ms. Maximum inward and outward currents were plotted against the applied voltage. Data interpretation was conducted with Origin Pro (OriginLab), and the second derivative was derived with Savitzky−Golay smooth (polynomial order 3, points of window: 30).

#### Bulk RNA sequencing

Total RNA was isolated from single hCOs, A375-MBMs, and 2D A375 cells (*n* = 5 per condition) using QIAshredder (QIAGEN, 79656) and the RNeasy Micro Kit (QIAGEN, 74004). RNA concentration was measured by the NanoDrop 1000 spectrophotometer (Thermo Fisher Scientific). RNA sequencing was performed at CeGAT GmbH with the library preparation kit TruSeq Stranded mRNA (Illumina) and NovaSeq 6000, 2x101bp. The reads were aligned to the Ensembl human reference genome (GRCh38) using STAR v.2.4 with default parameters.^64^ The overlap with annotated gene loci was counted with featureCounts v.1.5.1.^65^ Differential expression analysis was performed with DESeq2 (v.3.12)^66^ calling differentially expressed genes with a minimal 2-fold change and false discovery rate (FDR)-adjusted *p* < 0.05. Top regulated genes were identified using the Wald-statistic that is used by DESeq2. Gene lists were annotated using biomaRt (v.4.0).^67^ Gene set enrichment analysis (GSEA) was performed using the clusterProfiler package and STRING.^68^ Principal component analysis was performed using the top 500 regulated genes estimated by Wald-statistics.

#### Transmission electron microscopy

hCOs and A375-MBMs for ultrastructural analysis were first fixed in Karnovsky fixative (2.5% glutaraldehyde (EMS, 16400), 2% paraformaldehyde (EMS, 19200), 0.1 M sodium-cacodylate buffer (EMS,12310), 1% sucrose (EMS, 21600)) and afterward embedded (post-fixation with osmium tetroxide (EMS,19110), dehydration with graded ethanol (Sigma-Aldrich, 32221-M), infiltration with EPON (90 g (EMbed812) (EMS, 14900), 60 g DDSA (EMS, 13710), 40 g NMA (EMS, 19000), 3 g DMP-30 (EMS, 13600)) in the LYNX microscopy tissue processor (Reichert-Jung). The EPON blocks were polymerized for two days at 60°C. Semi-thin sections (0.75 μm) for the selection of relevant areas and ultra-thin sections (80 nm) were cut with an Ultracut S Microtome (Reichert-Jung) with diamond knives (DiATOME). The ultra-thin sections were contrasted with aqueous 2% uranyl acetate (Fluka, 94260) and 2% lead-citrate solution (Ultrostain 2) (Leica Biosystems, 16707235) for 10 min each. For the sample documentation, the side-mounted 2kx2k camera (TRS TRÖNDLE Restlichtverstärkersysteme) of a LEO 912AB electron-microscope (Zeiss) was used with the imageSP-software (TRS TRÖNDLE Restlichtverstärkersysteme).

#### Scanning electron microscopy

For high-resolution scanning electron microscopy, MBM models were fixed overnight at room temperature by the addition of equal volumes of 8% formaldehyde in D-PBS (Sigma-Aldrich, 252549, D8537) to the culture medium and washed three times with D-PBS. Fixed MBM models were transferred in a small droplet onto a 1 cm thick piece of PDMS (Dow, SYLGARD 184 Elastomer Kit) and lanced with a 150 μm thick stainless-steel needle (Golrisen). Subsequently, MBM models were dehydrated by a stepwise ethanol exchange, i.e., incubation in 25% ethanol in PBS, 50% EtOH in deionized (DI) water, 75% ethanol in DI water, and 100% ethanol (VWR International, 85651) for 10 min at room temperature each. Critical point drying was conducted using an Autosamdri-815 (Tousimi). Factory presets were used, purge time was 15 min. Dried MBM models were sputtered with 5 nm gold palladium (Leica Microsystems, EM ACE 200) from top and bottom, and stored at room temperature until imaging with a Crossbeam 550 (Zeiss).

#### CXCL10 ELISA

Cell supernatant was collected from hCOs and A375-MBM cultures at day 50 or day 56, and stored at −20°C. Chemokine concentration was determined using the Human CXCL10/IP10 Quantikine enzyme-linked immunosorbent assay (ELISA) kit (Bio-Techne, DIP100) according to the manufacturer’s instructions. The optical density was measured using the EnVision Multimode Plate Reader (Revvity). All measurements were performed in duplicates.

#### Flow cytometry

For tdTomato expression analysis, cells were fixed in BD Cytofix Fixation Buffer (BD Biosciences, 554722) for 20 min at room temperature and analyzed using FACSymphony A3. To confirm GFP expression, cells were fixed in FluoroFix Fixation Buffer (BioLegend, Cat# 422101) for 20 min and analyzed using Gallios 10C 3L Flow Cytometer (Beckmann Coulter). For phenotyping, cells were stained for NGFR (1:20, BioLegend, Cat# 345131) or isotype control (1:33, BioLegend, 400131) for 20 min at room temperature, fixed in FluoroFix Fixation Buffer (BioLegend, Cat# 422101) for 20 min, and subsequently washed with 1 × Permeabilization Wash Buffer (BioLegend, Cat# 421002). Intracellular marker Melan-A (1:40, Santa Cruz Biotechnology, Sc-20032) or isotype control (1:20, BioLegend, 400155) was stained for 30 min at room temperature. Labeled cells were recorded in FACS Buffer (2% FCS in D-PBS) using Gallios 10C 3L Flow Cytometer (Beckmann Coulter). Data were analyzed using the FlowJo software (BD Biosciences).

#### Anti-cancer approved library drug screen

Anti-cancer approved drug library (L2110-1-TM) was acquired from Hölzel Diagnostika Handels GmbH containing 313 compounds at a concentration of 10 mM in DMSO. We further included temsirolimus (Biomol, TGM-T2145) and GSK343 (Biomol, TGM-T6059) in the library. From drug library source plates, dilution plates containing 1 mM, 0.1 mM, or 0.001 mM compound concentration were prepared, and 8 μL of compound was transferred into Echo Qualified 384-Well Low Dead Volume Microplates. Library plates (Labcyte, 001–16128) were stored at −80°C. Final assay-ready plates (Greiner Bio-One,781091) were prepared using the Echo 550 liquid handler (Labcyte) to obtain a final compound concentration of 1 μM for single-point drug screening. 10 μM bortezomib (Hölzel Diagnostika Handels GmbH, TMO-T2399) was used as a positive control and 0.1% DMSO as a negative control on each plate. For drug validation, assay-ready plates with 10-point dose-response titration in 1:4 dilution steps starting from 50 μM were prepared. For 2D drug screen, single cell suspensions (300–2500 cells/well, depending on growth rate) of A375, MelDCC3, and MelDCC8 were seeded in 40 μL complete medium in assay-ready plates using MultiDrop Combi reagent dispenser (Thermo Fisher Scientific) and Standard Tube Cassettes (Steinle Labtechnology). For 3D drug screening, tumoroids were counted by live imaging using the Operetta Imaging System (Revvity) based on GFP signal in 96-well plates (Greiner Bio-One, 655090) with 10× objectives, non-confocal mode, 5% overlap, and analyzed using a building block-based algorithm in Harmony software (Revvity). 200 tumoroids per well were plated in assay-ready plates in 40 μL complete medium containing 0.005% GrowDex-T (Revvity, 200103002). Plates were sealed with Breathseal sealer (Greiner Bio-One, 676050) and incubated for 120 h. Sensitivity of 2D cultures toward compounds was assessed by ATPlite 1step Luminescence Assay System (Revvity, 6016736) following manufacturer’s instructions. MelDCC tumoroid viability was measured using CellTiter-Glo 3D Cell Viability Assay (Promega, G9682) according to the manufacturer’s instructions. Luminescence signals were measured with EnVision Multimode Plate Reader (Revvity). Values were normalized to corresponding DMSO controls. As a quality criterion, we calculated Z′ values for each assay plate. Data for the MelDCC11 cell line and immortalized melanocytes (NHEM) were included from S.M.-R., M.W.-K., C.A.K., K. Weidele, C. Werno, and S. Treitschke (unpublished data).

#### Drug sensitivity assay in 3D MBM models

For the validation of the primary drug screen, hit compounds were screened using 50-day-old A375-MBMs at 10 μM concentration for 72 h in comparison to the combination therapy of encorafenib and binimetinib. Compounds were selected from an in-house drug repurposing library based on.^43^ For dosing experiments of selinexor (Selleckchem, S7252) and DEBIO-0932 (MedChemExpress, HY-13469) in MBM models, either dose-response titration in 1:2 dilution steps starting from 10 μM or single compound concentrations were refreshed daily during six consecutive days. Control wells were treated with 0.1% DMSO. Compounds and concentrations were tested in duplicates or triplicates. All experiments were performed by full medium changes of organoid maintenance medium supplemented with compounds (100 μL/well) in final the dilution using multichannel pipettes. The viability of MBMs was measured by brightfield and fluorescence live imaging as described before. To measure caspase activation, we performed the Caspase-Glo 3/7 3D Assay (Promega, G8981) according to the manufacturer’s instructions. Caspase-Glo reagent was added in equal amounts, mixed on a shaker (30 s, 60 rpm), and incubated for 30 min at room temperature. Luminescence was recorded using the EnSight multimode plate reader (Revvity). Viability values were normalized to corresponding DMSO controls. Samples referred to as prolonged A375-MBM cultures correspond to the DMSO control samples at day 56.

#### Drug sensitivity assay in hCOs

hCOs were treated with compounds as described above in parallel to corresponding A375-MBMs. For the assessment of hCO viability, we performed CellTiter-Glo 3D Cell Viability Assay (Promega, G9682) according to the manufacturer’s instructions. For this, the CellTiter-Glo reagent was added in equal amounts, and cell lysis was induced by repetitive pipetting and repetitive shaking (5 min, 60 rpm). Lysates were transferred in a ratio of 1:10 to D-PBS in white-bottom microplates (Greiner Bio-One, 655083) and additionally incubated with equal amount of the CellTiter-Glo reagent, first on a shaker (5 min, 60 rpm) and then for another 25 min at room temperature. Luminescence was recorded using the EnSight multimode plate reader (Revvity). Viability values were normalized to corresponding DMSO controls.

#### Impedance measurements

To monitor the integrity of the *in vitro* BBB over time, we used impedance spectroscopy, enabling simultaneous measurement of transendothelial electrical resistance (TEER) and cell layer capacitance (C_cl_). hiPSC-derived BCECs were seeded onto inserts and placed in a 24-well cell module of a cellZscope device (nanoAnalytics) inside an incubator at 37°C with 5% CO_2_. TEER and C_cl_ values were extracted from the impedance spectra using equivalent circuit modeling as implemented in the cellZscope software. Both values were assessed every hour for a duration of 96 h.

#### *In vitro* blood-brain barrier permeability assay

At day 10 of differentiation, hiPSC-derived BCECs were used to investigate the BBB permeation of selinexor and DEBIO-0932. We compared their transport properties to reference substances with varying BBB permeation: Carbamazepine (quick permeation; Sigma-Aldrich, C4024), atenolol (slow permeation; Sigma-Aldrich, A7655), and verapamil (considered medium permeation, as its classification varies by study and model used; Sigma-Aldrich, V4629).^72^ Compounds were diluted in EC medium to a concentration of 10 μM and applied to the donor compartment, which is the apical (A) compartment for A-to-B transport or the basolateral (B) compartment for B-to-A transport. After incubating the BCECs with compounds for 1 h at 37°C on an orbital shaker set to 100 rpm, medium samples from both apical and basolateral compartments were collected for downstream analysis. To monitor monolayer integrity during the experiment, 10 μM fluorescein solution (Sigma-Aldrich, F6377) was added together with the test compounds in each apical compartment at a concentration of 10 μM. The concentration of the fluorescent tracer molecule was measured using a fluorescent plate reader (excitation: 490 nm, emission: 525 nm; Infinite M1000 Pro, TECAN), and its permeability coefficient was calculated according to the clearance principle, as described previously,^73^ to confirm the integrity of the BBB during compound treatment. The samples for liquid chromatography with tandem mass spectrometry (LC-MS/MS) measurement were stored at −80°C until analysis. Apparent permeability (*P*_app_) values were calculated from peak areas using the following equation, as previously described^74^:

$$P_{app}=\frac{\Delta Q}{\Delta t}\times \frac{1}{A\times \left(\frac{C_{D}+C_{0}}{2}\right)}$$

Where ΔQ is the amount of the compound permeated into the acceptor compartment as determined by the response (= peak area) of compound in the acceptor compartment at the end of the incubation time. A is the surface area of the insert membrane (0.34 cm^2^), C_0_ is the initial nominal compound concentration, C_D_ is the response in the donor compartment at the end of the incubation time, and t the incubation time.

The efflux ratio (ER) is calculated as follows, with ER > 2 indicating active efflux:

$$ER=\frac{P_{app}\left(BA\right)}{P_{app}\left(AB\right)}$$

Where *P*_app_ (A-B) refers to the permeability in the direction of apical to basolateral, and *P*_app_ (B-A) refers to the permeability in the direction of basolateral and apical.

Unless stated otherwise, only assays with recovery rates (mass balance) between 65% and 135% were considered. Recovery was determined with the following equation:

$$Recovery\;\left(\%\right)=\frac{C_{A}+C_{D}}{C_{0}}\;x\;100$$

Where C_A_ and C_D_ are the responses of the compound determined in the acceptor and donor compartments at the end of the incubation time, respectively. C_0_ is the initial nominal compound concentration.

#### LC-MS/MS sample preparation and analysis

For LC-MS/MS analysis, the samples from the BBB permeability assays were thawed and thoroughly mixed for 5 min at 600 rpm on an orbital microtiter plate shaker (IKA MTS 4) and 5x up- and down pipetting. Donor samples were diluted 1:5 with assay buffer (EC medium), and all samples were mixed with 100% ethanol containing 25 nM Carbutamid (AbbVie, in-house synthesis) as internal LC-MS/MS process control. The samples were centrifuged at 3000 x *g* for 30 min, and the supernatants were taken for measurement.

LC-MS/MS analysis was performed using an Acquity UPLC (Waters) coupled with a tandem mass spectrometer Sciex 6500+ (Sciex) operating in multiple reaction monitoring (MRM). Samples were separated with the BEH C18 column (30 mm × 2.1 mm, 1.7 μm; Waters, 186002349) at 50°C using a two-step gradient elution with water (mobile phase A, VWR International, 84867.290) and acetonitrile (mobile phase B, VWR International, 83640.290), both acidified with 0.1% formic acid. The run time of chromatographic separation was a linear 1.1 min gradient, starting with 95% mobile phase A and peaking with 95% mobile phase B. In the case of selinexor, mobile phase A was replaced with 25 mM ammonium bicarbonate (Sigma-Aldrich, 5.33005) supplemented with 25 mM ammonium hydroxide (Honeywell International, 30501-1L). The mass spectrometer was operating in positive electrospray ionization (ESI) mode. Data acquisition and analysis were performed using Analyst 1.7.2 (Sciex). Results were reported and further used for calculation as peak area (response).

### Quantification and statistical analysis

Statistical analysis was performed with GraphPad Prism 10.2.2 Software. Unless otherwise indicated, data are presented as mean with the standard error of the mean and individual data points plotted. Replicates were selected by random sampling of each hCO or MBM batch. Asterisks represent the significance as follows: ∗∗∗: *p* < 0.001, ∗∗: *p* < 0.01, ∗: *p* < 0.05 and ns: *p* ≥ 0.05. Information on statistical analysis and number of replicates is stated in the respective figure legend. Dose-response curves and IC_50_ values were calculated and plotted based on a sigmoidal dose-response equation. As maximum efficacy (E_max_), we calculated the differences between the highest and lowest points of the curve. GraphPad Prism was used to perform Pearson correlation analysis to compare screening plates and coefficient of variation analyses to determine intra-batch variability.

## Supplemental information

Document S1. Figures S1–S4

Document S2. Article plus supplemental information
