# Supplementary material for: Cortical organoid-derived models of the melanoma brain metastatic niche enable prioritization of cancer-targeting drugs
Source: Cell Rep Methods. 2025 Nov 14;5(12):101236. doi: 10.1016/j.crmeth.2025.101236 (PMC12859518; doi:10.1016/j.crmeth.2025.101236)
Supplement: Document S1. Figures S1–S4 [file mmc1.pdf]

Cell Reports Methods, Volume *5*

Supplemental information

Cortical organoid-derived models of the melanoma brain metastatic niche enable prioritization of cancer-targeting drugs

Kim Krieg, Silvia Materna-Reichelt, Tobias Naber, Fatima-Zahra Rachad, Pia Kauven, Arjen Weller, Undine Haferkamp, Annika Wittich, Andrea Zaliani, Marcel S. Woo, Mark Walkenhorst, Malte Siegmund, Jann Harberts, Robert Zierold, Robert Blick, Christian Conze, Patricia Muschong, Dominik Miltner, Manuel A. Friese, Mario Mezler, Heiko Siegmund, Katja Evert, Susanne Krasemann, Nataša Stojanović Gužvić, Christoph A. Klein, Melanie Werner-Klein, Joachim Wegener, and Ole Pless

Supplemental Information

Supplemental figures and legends

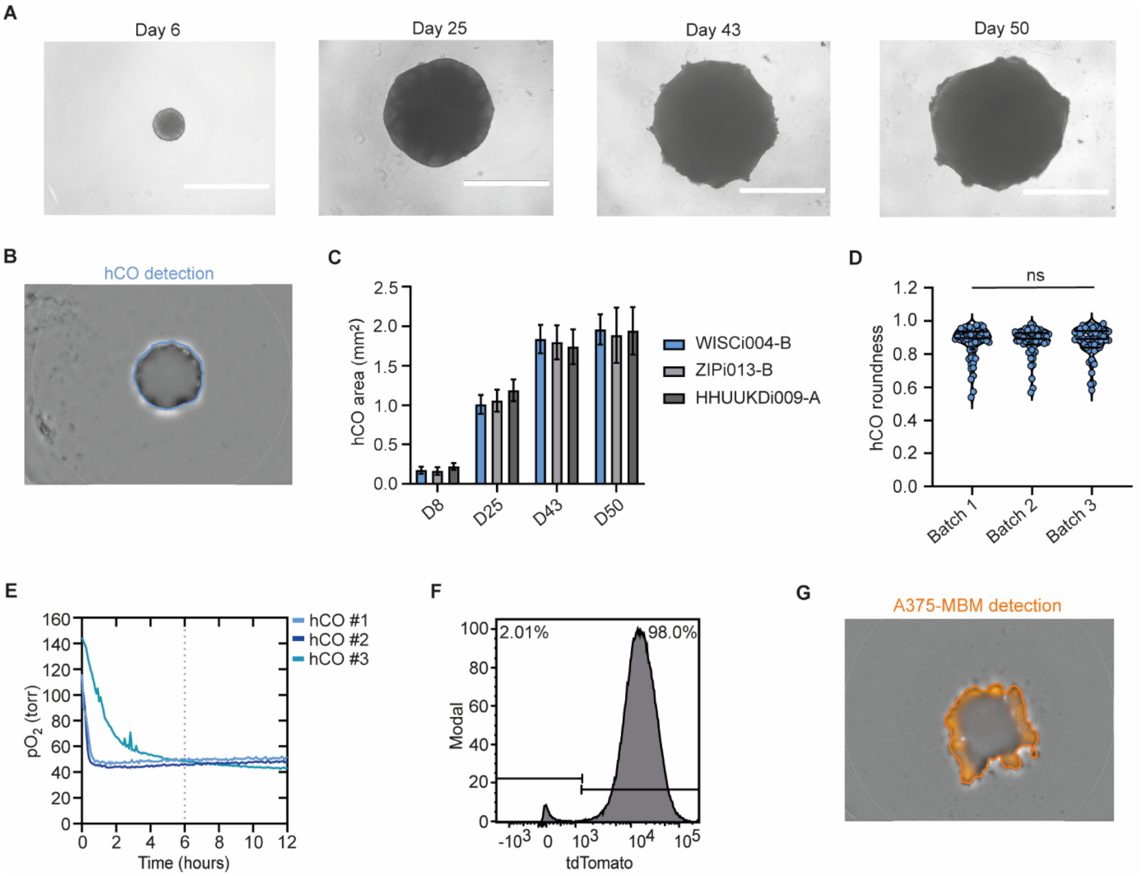

Figure S1: Generation of A375-MBMs using reproducible hCOs and a A375-tdTomato fluorescent reporter cell line, related to Figure 1.

(A) Microscopic brightfield images present hCOs at different time points during differentiation. Scalebars represent 1 mm.

(B) Illustration of spheroid detection analysis of hCOs based on brightfield imaging. Blue line indicates the considered hCOs area for morphology analysis.

(C) Comparable increase of organoid area during differentiation of hCOs derived from hiPSC lines WISCi004-B, ZIPi013-B and HHUUKDi009-A ($n \geq 57$ hCOs from each batch per time point). Data are presented as mean ± SEM.

(D) Consistent mean hCO roundness at day 50 ($n \geq 57$ hCOs per batch). Data are presented with median and lower/upper quartile. Statistical analysis was performed using one-way ANOVA, followed by Tukey's multiple comparisons test. ns, not significant.

(E) Time-resolved analysis of partial oxygen pressure (pO_2) of 3 hCOs from independent batches after seeding on the oxygen sensor foil.

(F) Flow cytometry analysis of reporter expression in A375-tdTomato after lentiviral transduction and antibiotic selection.

(G) Illustration of spheroid detection analysis of A375-MBM based on brightfield and fluorescence imaging. Orange line indicates the considered A375-MBM area for analysis of the fluorescence intensity.

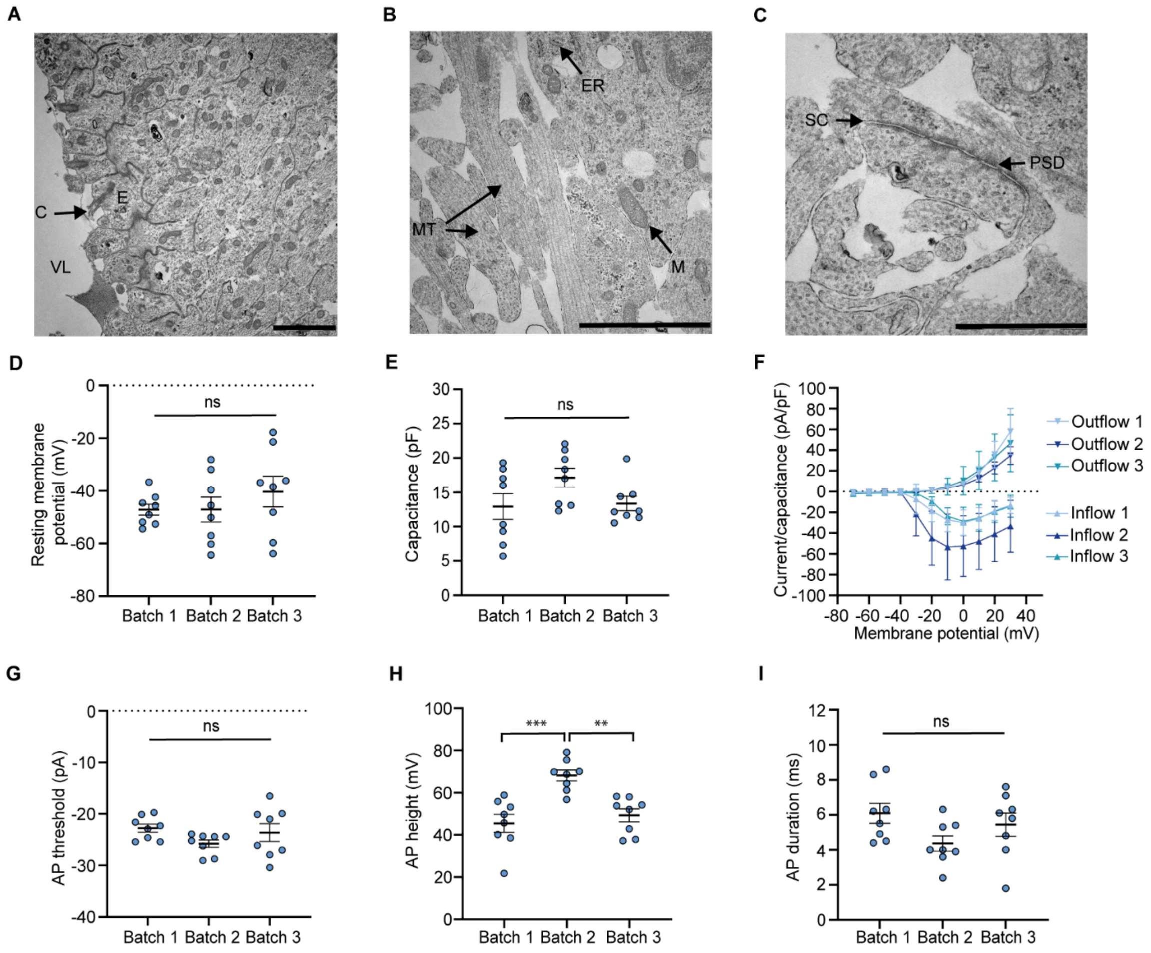

Figure S2: Tissue integrity and functionality of hCO-derived neurons, related to Figure 2.
(A) Electron microscopy image of ventricular zone-like structure in 50-days old hCOs with ventricular lumen (VL) surrounded by an ependymal cell layer (E). Arrow point at cilia (C). Scale bar represents 2 μm.
(B) Ultrastructural analysis of microtubule (MT) enriched neuronal-like structures. Arrows also point to mitochondria (M) and endoplasmic reticulum (ER). Scale bar represents 2 μm.
(C) Visualization of synapse-like structures using electron microscopy with arrows pointing at cleft (SC) and postsynaptic density (PSD). Scale bar represents 1 μm.
(D-E) Comparison of passive membrane properties of hCO-derived neurons ($n = 8$) across independent batches using patch clamp techniques. Data are presented as mean ± SEM. Statistical analysis was performed using one-way ANOVA, followed by Tukey's multiple comparisons test. ns, not significant.
(F) In- and outward currents of three batches ($n = 8$ neurons). Data are presented as mean ± SEM. Currents were plotted against the applied membrane potential and normalized to the capacitance.
(G-I) Quantification of action potential (AP) shape during electrical depolarization ($n = 8$ neurons). Data are presented as mean ± SEM. Statistical analysis was performed using one-way ANOVA, followed by Tukey's multiple comparisons test. ns, not significant, ** $p < 0.01$, *** $p < 0.001$.

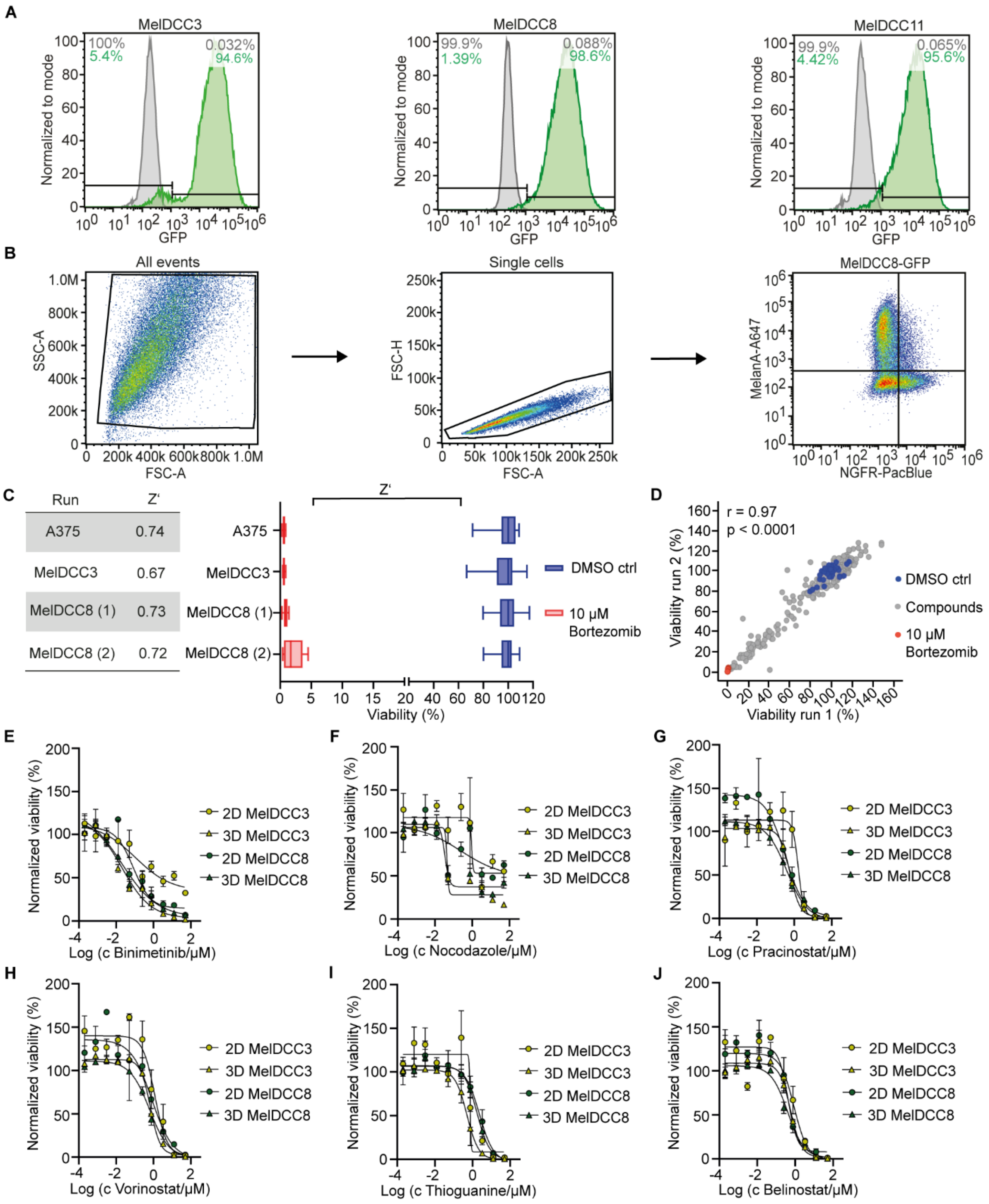

Figure S3: Anti-cancer drug screen using patient-derived MelDCCs, related to Figure 4.

(A) Flow cytometry analysis of GFP expression in parental (grey) and stably transduced GFP-reporter (green) MelDCC lines.

(B) Gating scheme for marker expression analysis of NGFR and Melan-A by flow cytometry. (C) Z' calculation for all screening plates using DMSO control and 10 µM bortezomib as positive control. (D) Pearson correlation of replicated screening runs of MelDCC8.

(E-J) Concentration-response titration of hit compounds based on ATP-based cellular viability of 2D and tumoroids (3D) cell cultures (n = 2 - 3 experiments). Data are presented as mean ± SEM. (E) Binimetinib IC_{50}(2D MelDCC3): 0.56 µM; IC_{50}(3D MelDCC3): 0.86 µM; IC_{50}(2D MelDCC8): 0.4 µM; IC_{50}(3D MelDCC8): 0.42 µM. (F) Nocodazole IC_{50}(2D MelDCC3): 0.05 µM; IC_{50}(3D MelDCC3): 0.93 µM; IC50(2D MelDCC8): 0.03 µM; IC_{50}(3D MelDCC8): 0.25 µM. (G) Pracinostat IC_{50}(2D MelDCC3): 0.58 µM; IC_{50}(3D MelDCC3): 1.63 µM; IC_{50}(2D MelDCC8): 0.40 µM; IC_{50}(3D MelDCC8): 0.32 µM. (H) Vorinostat IC_{50}(2D MelDCC3): 0.67 µM; IC_{50}(3D MelDCC3): 1.18 µM; IC_{50}(2D MelDCC8): 0.62 µM;

IC_{50}(3D MelDCC8): 0.81 μM. (I) Thioguanine IC_{50}(2D MelDCC3): 0.5 μM; IC_{50}(3D MelDCC3): 0.74 μM; IC_{50}(2D MelDCC8): 1.72 μM; IC_{50}(3D MelDCC8): 2.29 μM. (J) Belinostat IC_{50}(2D MelDCC3): 0.56 μM; IC_{50}(3D MelDCC3): 0.86 μM; IC_{50}(2D MelDCC8): 0.40 μM; IC_{50}(3D MelDCC8): 0.42 μM.

A

Cell line	Cell type	Sex
WISCi004-B	hiPSC	Female
ZIPi013-B	hiPSC	Female
HHUUKDi009-A	hiPSC	Male
A375	Melanoma cell	Female
MelDCC3	DCC	Male
MelDCC8	DCC	Male

MBM model nomenclature	hiPSC (hCO scaffold)	Sex
A375-MBM 1	WISCi004-B	Female
A375-MBM 2	ZIPi013-B	Female
A375-MBM 3	HHUUKDi009-A	Mixed
MelDCC8-MBM 1	WISCi004-B	Mixed
MelDCC8-MBM 2	ZIPi013-B	Mixed
MelDCC8-MBM 3	HHUUKDi009-A	Male

B

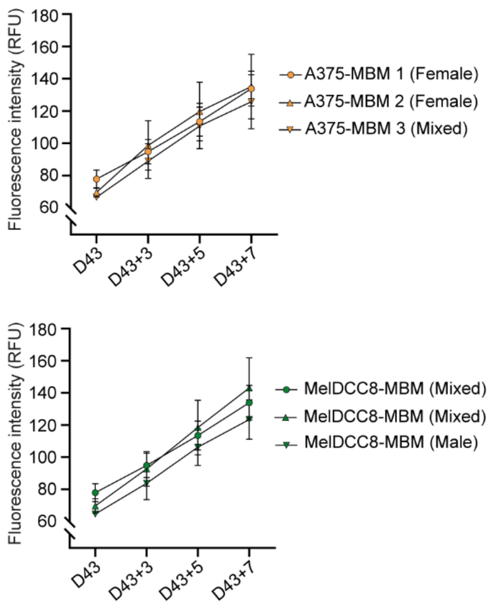

Figure S4: Validation of the MBM model workflow using patient-derived MelDCCs in combination with hCOs derived from sex-matched hiPSC donors, related to Figure 5.

(A) Upper panel: Overview of hiPSC and cancer cell line information, including sex. Superscript numbers refer to the corresponding references. Lower panel: Overview of female, male and mixed-sex A375-MBM and MelDCC8-MBM models.

(B) Comparative time course analysis of female, male and mixed-sex A375- and MelDCC8-MBM models demonstrates similar growth pattern (n = 30 samples each batch). Data are presented as mean ± SD. Data for A375-MBM 1 and MelDCC8-MBM 1 (n = 6 independent batches each) are presented as mean ± SEM.
